# A base editing strategy using mRNA-LNPs for *in vivo* correction of the most frequent phenylketonuria variant

**DOI:** 10.1016/j.xhgg.2023.100253

**Published:** 2023-11-02

**Authors:** Dominique L. Brooks, Madelynn N. Whittaker, Hooda Said, Garima Dwivedi, Ping Qu, Kiran Musunuru, Rebecca C. Ahrens-Nicklas, Mohamad-Gabriel Alameh, Xiao Wang

**Affiliations:** 1 Cardiovascular Institute, Perelman School of Medicine at the University of Pennsylvania, Philadelphia, PA 19104, USA; 2 Division of Cardiovascular Medicine, Department of Medicine, Perelman School of Medicine at the University of Pennsylvania, Philadelphia, PA 19104, USA; 3 Department of Genetics, Perelman School of Medicine at the University of Pennsylvania, Philadelphia, PA 19104, USA; 4 Department of Bioengineering, University of Pennsylvania, Philadelphia, PA 19104, USA; 5 Department of Bioengineering, George Mason University, Fairfax, VA 22030, USA; 6 Division of Infectious Diseases, Department of Medicine, Perelman School of Medicine at the University of Pennsylvania, Philadelphia, PA 19104, USA; 7 Metabolic Disease Program, Division of Human Genetics, Department of Pediatrics, Children’s Hospital of Philadelphia, Philadelphia, PA 19104, USA; 8 Department of Pediatrics, Perelman School of Medicine at the University of Pennsylvania, Philadelphia, PA 19104, USA; 9 Department of Pathology and Laboratory Medicine, Children’s Hospital of Philadelphia, Philadelphia, PA 19104, USA; 10 Department of Pathology and Laboratory Medicine, Perelman School of Medicine at the University of Pennsylvania, Philadelphia, PA 19104, USA

**Keywords:** base editing, CRISPR, gene editing, genome editing, inborn error of metabolism, metabolic disease, phenylketonuria, prime editing, rare disease

## Abstract

The c.1222C>T (p.Arg408Trp) phenylalanine hydroxylase (*PAH*) variant is the most frequent cause of phenylketonuria (PKU), an autosomal recessive disorder characterized by accumulation of blood phenylalanine (Phe) to neurotoxic levels. Here we devised a therapeutic base editing strategy to correct the variant, using prime-edited hepatocyte cell lines engineered with the c.1222C>T variant to screen a variety of adenine base editors and guide RNAs *in vitro*, followed by assessment in c.1222C>T humanized mice *in vivo*. We found that upon delivery of a selected adenine base editor mRNA/guide RNA combination into mice via lipid nanoparticles (LNPs), there was sufficient *PAH* editing in the liver to fully normalize blood Phe levels within 48 h. This work establishes the viability of a base editing strategy to correct the most common pathogenic variant found in individuals with the most common inborn error of metabolism, albeit with potential limitations compared with other genome editing approaches.

## Teaser

The *PAH* c.1222C>T variant is the most frequent variant reported in phenylketonuria (PKU) individuals. Here, the authors use base editing, delivered by mRNA-lipid nanoparticle technology, to directly correct the variant in the liver in humanized PKU mice and definitively treat the disease.

## Main text

Phenylketonuria (PKU [MIM: 261600]) is a disorder of phenylalanine (Phe) metabolism wherein deficiency of phenylalanine hydroxylase (PAH) results in elevated blood Phe levels. Optimal management of PKU requires strict, lifelong monitoring and control of blood Phe levels to maintain them within the recommended range of 120–360 μmol/L.^1^ When not adequately treated, blood Phe levels can exceed 1200 μmol/L, and as a result, PKU individuals can develop irreversible neurological impairment and neuropsychiatric issues. Although there are several treatment options to regulate blood Phe levels within the recommended range––including a low-Phe diet, an oral medication that serves as a cofactor of PAH (sapropterin), and a daily injectable enzyme substitution therapy (pegvaliase)—more than 70% of adults with PKU are noncompliant with treatment guidelines due to challenges associated with adherence and therapy responsiveness.^2^

The most frequently occurring pathogenic *PAH* variant worldwide is the c.1222C>T (p.Arg408Trp) variant (RefSeq: NM_000277.3), particularly prevalent in European countries and the United States.^3^ We have found in a parallel study^4^ being simultaneously published in *The American Journal of Human Genetics* that most individuals with *PAH* c.1222C>T variants experience chronic, severe Phe elevations, reflecting in part the limitations of the existing treatment options.^5^^,^^6^ Genome editing offers the potential of a one-time curative therapy to permanently normalize blood Phe levels. Base editing is particularly attractive because it can precisely and efficiently correct pathogenic variants.^7^^,^^8^ In a recent study, we found that base editing could rapidly (within 48 h) and definitively treat a humanized mouse model of PKU with the *PAH* c.842C>T (p.Pro281Leu) variant when intravenously delivered in the form of mRNA and guide RNA (gRNA) encapsulated in lipid nanoparticles (LNPs) targeting the liver, where *PAH* mRNA is specifically expressed.^9^ As little as 10% correction of the gene is sufficient to normalize blood Phe levels.

**Figure 1:**
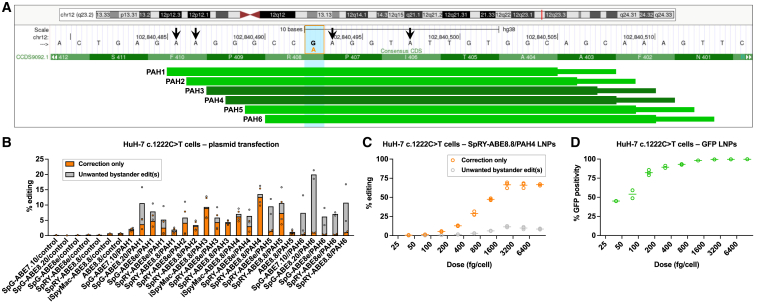
Base editing to correct *PAH* c.1222C>T variant in human hepatocytes *in vitro* (A) Schematic of the genomic site of the *PAH* c.1222C>T variant, adapted from the UCSC Genome Browser (GRCh38/hg38). The vertical blue bar outlined by the orange box indicates the G altered to A (in orange) by the variant on the antisense strand. The arrows indicate the sites of potential bystander editing. The horizontal green bars indicate protospacer (thick) and PAM (thin) sequences targeted by the PAH1 through PAH6 gRNAs. (B) Corrective *PAH* c.1222C>T editing (determined from genomic DNA) following transfection of cells with plasmids encoding adenine base editor/gRNA combinations (n = 2 biological replicates, one each from two *PAH* c.1222C>T homozygous HuH-7 cell lines; controls, n = 1), calculated as the proportion of aligned sequencing reads with the indicated type of edits. “Correction only” refers to reads in which the c.1222C>T adenine variant is edited to guanine, with or without base editing of the adjacent synonymous adenine, with no base editing of any other adenines; “unwanted bystander editing” refers to reads in which the c.1222C>T adenine variant is edited to guanine, along with base editing of one or more nonsynonymous adenines. (C) Dose-response study with *PAH* c.1222C>T homozygous HuH-7 cells treated with SpRY-ABE8.8/PAH4 LNPs (n = 3 biological replicates). (D) Dose-response study with *PAH* c.1222C>T homozygous HuH-7 cells treated with GFP LNPs (n = 2 to 3 biological replicates).

In principle, because the *PAH* c.1222C>T variant results from a C→T change on the sense strand, the variant is amenable to correction by an adenine base editor introducing an A→G change at the same position on the antisense strand. However, upon inspection of the genomic site of the variant (Figure 1A), two substantial impediments to therapeutic base editing become evident. First, the position of the target adenine does not lie within the editing window (roughly from positions 3 to 9 of the protospacer sequence) for any protospacer that has an NGG protospacer-adjacent motif (PAM), the preferred PAM for standard *Streptococcus pyogenes* Cas9 (SpCas9)-containing editors. Accordingly, one would need to use either SpCas9 variants with altered PAM preferences or non-Sp Cas9 proteins with non-NGG PAM preferences, likely with reduced editing efficiency even if there is optimal spacing of the target adenine within the editing window. Second, there are four nearby non-target adenines that could potentially be edited in conjunction with the target adenine, resulting in bystander editing. The adenine in the adjacent position downstream of the target adenine, if converted to guanine, would represent a synonymous edit (codon CCT to codon CCC, both encoding proline) unlikely to have a functional consequence. In contrast, the adenine located five positions downstream of the target adenine, if converted to guanine, would represent a nonsynonymous edit (codon ATA to codon ACA) resulting in the *PAH* c.1217T>C (p.Ile406Thr) variant, which has been reported to occur in PKU individuals and thus is likely to compromise PAH function.^10^^,^^11^ Similarly, the adenines located six and seven positions upstream of the target adenine, if singly or both converted to guanine, would represent nonsynonymous edits (codon TTC to codon CTC, TCC, or CCC) that would change the phenylalanine in amino acid position 410 to leucine, serine, or proline. Thus, achieving the desired on-target editing without undesired bystander editing in either direction could prove challenging.

In our parallel study,^4^ we had already used prime editing to generate clonal HuH-7 human hepatoma cell lines (as a proxy for human hepatocytes) homozygous for the *PAH* c.1222C>T variant. Using two of the clonal lines, we evaluated an array of adenine base editors and gRNAs for editing activity at the site of the variant, assessing for both on-target editing and unwanted bystander editing. We used four different Cas9 variants: standard SpCas9 (which prefers NGG PAMs but can also engage NAG and NGA PAMs, albeit with less activity), iSpyMac (which prefers NAA PAMs),^12^ SpG (which generally engages NGN PAMs),^13^ and SpRY (which being near-PAMless can engage a broad range of sequences).^13^ We used four different adenosine deaminase domains: ABE7.10 (seventh-generation, less activity),^8^ ABE8e (eighth-generation, more activity, broadest editing window),^14^ ABE8.20 (eighth-generation, more activity, intermediate editing window),^15^ and ABE8.8 (eighth-generation, more activity, narrowest editing window).^15^ We used six different gRNAs (termed PAH1 through PAH6) tiling the site of the variant, with distinct PAMs (AGC, GCA, CAA, AAA, AAG, and AGT, respectively) (Figure 1A). Via plasmid transfection, we tested a subset of combinations of Cas9 variant, adenosine deaminase domain, and gRNA, tailored to the position of the variant within the editing window and to the available PAM (Figures 1B and S1). To serve as a reference for transfection efficiency, we transfected a plasmid encoding green fluorescent protein (GFP) and observed mean 85% GFP positivity as determined by flow cytometry. In c.1222C>T homozygous HuH-7 cells, SpRY-ABE8.8/PAH4 yielded the best combination of higher on-target editing and lower bystander editing, reflecting that PAH4 places the variant adenine in the middle of the window (protospacer position 5), ABE8.8 has a narrow window that limits bystander editing on either side of the variant adenine, and SpRY provides more activity than iSpyMac with the same PAH4 gRNA with its AAA PAM (in contrast to the PAH3 gRNA, for which iSpyMac outperformed SpRY). We formulated LNPs with SpRY-ABE8.8 mRNA and synthetic PAH4 gRNA and performed a dose-response experiment in c.1222C>T homozygous HuH-7 cells, observing as high as mean 80% overall editing (with as high as mean 12% unwanted bystander editing) at the highest LNP doses and a half maximal effective concentration (EC_50_) of 750 fg/cell (Figures 1C and S2). To serve as a reference for transfection efficiency, we also formulated LNPs with GFP mRNA and performed a similar dose-response experiment (Figure 1D).

To test the SpRY-ABE8.8/PAH4 combination *in vivo*, we used CRISPR-Cas9 targeting in mouse embryos to generate a humanized PKU model with the *PAH* c.1222C>T (p.Arg408Trp) variant (hereafter referred to as R408W mice) in the C57BL/6J background, in which we replaced a small portion of the endogenous mouse *Pah* exon 12 with the orthologous human sequence spanning the PAH4 gRNA protospacer/PAM sequences and containing the c.1222C>T variant (Figure S3A). Upon breeding the humanized c.1222C>T allele to homozygosity, we observed phenotypes consistent with PKU, including elevated blood Phe levels, mild hypopigmentation (resulting from reduced melanin synthesis due to decreased tyrosine levels because of deficient PAH activity), and reduced body weight (Figures S3B and S3C). (In our parallel study,^4^ we generated a different homozygous R408W mouse model via homologous recombination in mouse embryonic stem cells, replacing the entirety of *Pah* exon 12 as well as flanking intronic regions with the orthologous human sequence, but we did not use that alternative model for this base editing study.).

**Figure 2:**
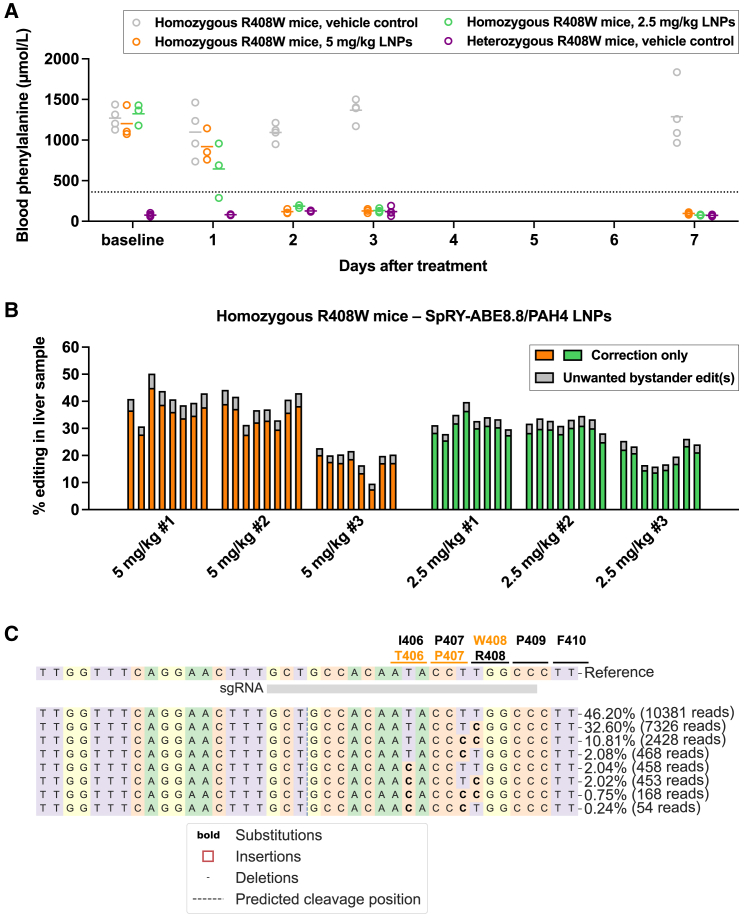
Base editing to correct *PAH* c.1222C>T variant in humanized mice (A) Changes in blood phenylalanine levels in homozygous PKU mice following treatment with 5-mg/kg dose of SpRY-ABE8.8/PAH4 LNPs (n = 3 animals) or with 2.5-mg/kg dose of LNPs (n = 3 animals), comparing levels at various timepoints up to 7 days following treatment to levels in vehicle-treated homozygous PKU control (n = 4 animals) and vehicle-treated heterozygous non-PKU control (n = 4 animals) age-matched (approximately 8 weeks of age) colonymates (one blood sample per time point). (B) Corrective *PAH* c.1222C>T editing (determined from genomic DNA) in each of eight liver samples (two samples each from the four lobes) from each treated mouse, calculated as the proportion of aligned sequencing reads with the indicated type of edits. “Correction only” refers to reads in which the c.1222C>T adenine variant is edited to guanine, with or without base editing of the adjacent synonymous adenine, with no base editing of any other adenines; “unwanted bystander editing” refers to reads in which the c.1222C>T adenine variant is edited to guanine, along with base editing of one or more nonsynonymous adenines. (C) Standard CRISPResso output for a liver sample from the LNP-treated homozygous PKU mouse with the highest level of editing. The codons in the vicinity of the c.1222C>T variant site are indicated; the top-listed amino acid is the baseline identity of the codon, and the bottom-listed amino acid is the one that results from base editing of the adenine in the codon. Lines in graphs = mean values.

We treated homozygous R408W PKU mice, approximately 8 weeks of age and with baseline blood Phe levels in the 1,000–1,500 μmol/L range, with SpRY-ABE8.8/PAH4 LNPs at two dose levels. PKU mice that received a single 5-mg/kg LNP dose experienced normalization of blood Phe levels by 48 h after treatment (mean 118 μmol/L, 90% reduction from baseline), and PKU mice that received a single 2.5-mg/kg LNP dose had substantial, though somewhat less, reduction of blood Phe levels by 48 h after treatment (mean 185 μmol/L, 86% reduction from baseline) (Figure 2A). All mice in both dose groups achieved blood Phe levels less than 125 μmol/L by 7 days after treatment. Vehicle-treated age-matched homozygous R408W PKU mice maintained elevated blood Phe levels during the same time course, and vehicle-treated age-matched heterozygous R408W non-PKU mice generally had blood Phe levels less than 125 μmol/L. There were no alanine aminotransferase (ALT) abnormalities in any of the mice over the same time period, with slight rises in aspartate aminotransferase (AST) levels at 1 day after treatment only in the mice that received the 5-mg/kg LNP dose, remaining within the normal range (Figures S4A and S4B). Out of a panel of 13 cytokines and chemokines, LNP treatment resulted in transient increases in C-X-C motif chemokine ligand 1, tumor necrosis factor-α, monocyte chemoattractant protein 1, interleukin (IL)-1β, interferon inducible protein 10, interferon-α, and IL-6 relative to vehicle treatment at 4 h after treatment, with resolution by 24 h after treatment (Table S1). Upon necropsy at 1 week after treatment, next-generation sequencing of genomic DNA from whole-liver samples to determine corrective editing activity showed mean 29% desired on-target editing and 4% undesired bystander editing in the higher-dosed mice and mean 26% desired on-target editing and 3% undesired bystander editing in the lower-dosed mice (Figures 2B and 2C). Liver histology showed no evidence of pathology (Figure S4C).

**Figure 3:**
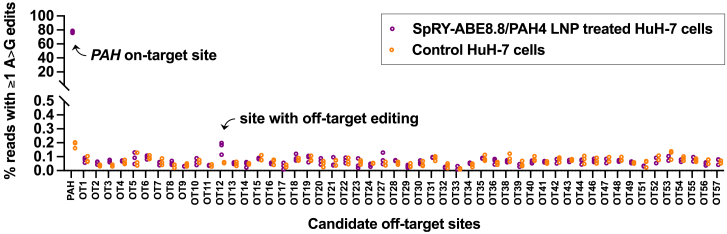
Assessment of off-target editing On-target or off-target editing at top *in silico*-nominated candidate sites calculated as the proportion of aligned sequencing reads with ≥1 adenine base edited to guanine within the editing window at each site in *PAH* c.1222C>T homozygous HuH-7 cells that underwent treatment with SpRY-ABE8.8/PAH4 LNPs at a dose of 10,000 fg/cell (n = 3 treated and 3 untreated biological replicates), the highest dose shown in Figure 1C. Sites with unsuccessful sequencing are omitted. Refer to Table S2 for candidate site sequences and numerical values.

A potential liability of the use of a SpRY Cas9 variant is a higher burden of off-target editing due to its near-PAMless nature allowing it to engage a far broader range of genomic sites than standard Cas9. To evaluate off-target editing by SpRY-ABE8.8/PAH4, we generated a list of 57 candidate genomic sites nominated by *in silico* prediction based on sequence similarity to the on-target *PAH* site—including sites with up to one protospacer mismatch plus up to two DNA or RNA bulges, or with up to two protospacer mismatches with no bulges, with no constraint on the PAM sequence. Next-generation sequencing of targeted PCR amplicons from genomic DNA extracted from SpRY-ABE8.8/PAH4 LNP-treated c.1222C>T homozygous HuH-7 cells, versus control cells, revealed just one site with very low off-target base editing (net editing of 0.11%), within a very large intron of *ILRAPL2* and unlikely to be of biological significance (Figure 3).

In our parallel study,^4^ we used an optimized prime editing configuration, delivered via dual adeno-associated viral (AAV) vectors, to correct the *PAH* c.1222C>T variant in humanized mice. A high dose of AAV treatment resulted in mean ≈40% corrective editing and no bystander editing, a better result than with SpRY-ABE8.8/PAH4 LNPs as reported in this study. To perform a head-to-head comparison between base editing and prime editing mediated by LNP delivery, we formulated LNPs with PEmax mRNA, synthetic prime editing guide RNA (pegRNA), and synthetic nicking guide RNA (ngRNA), analogous to the lead pegRNA/ngRNA combination used in the dual AAV configuration.^4^ Upon treatment of homozygous R408W PKU mice with a 5-mg/kg dose of these LNPs, there were no significant changes in blood Phe levels, and there was minimal whole-liver editing (<1%) upon necropsy (Figure S5). Possible reasons for the lack of efficacy of the prime editing LNPs made in the same way as the base editing LNPs include the need to synthesize the substantially longer mRNA (PEmax, ≈6.7 kb) and pegRNA (120 nucleotides) compared with the mRNA (SpRY-ABE8.8, ≈5.0 kb) and standard gRNA (100 nucleotides) used for base editing, which presents challenges of scale and purity; the need to encapsulate the larger mRNA along with two guide RNAs rather than one guide RNA within LNPs; slower kinetics of prime editing compared with base editing, which might require more prolonged expression of the editor in cells than that provided by the use of standard mRNA-LNPs; and reduced affinity of the pegRNA for the prime editing protein, due to auto-inhibition of the pegRNA.^16^ While prime editing has more flexibility of site selection and avoids the bystander editing observed with base editing, its dependence on high doses of AAV vectors for effective *in vivo* delivery currently makes it a less favorable therapeutic option than LNP-mediated base editing, an approach that is already being evaluated in clinical trials.^17^

We acknowledge the limitations of this study as well as the base editing approach reported here. We did not evaluate all possible combinations of the existing catalogs of PAM-altered Cas9 variants, adenosine deaminase domains, and gRNAs, and there likely are combinations that would have equal or better corrective editing efficiency while also having less bystander editing and more favorable off-target profiles, making them more credible therapeutic candidates. Alternative Cas9 variants include engineered SpCas9 variants preferring NGA PAMs,^18^ NGCG PAMs,^18^ NGK PAMs,^19^ NGN PAMs,^20^ or NRNH PAMs,^21^ as well as a wide variety of naturally occurring and engineered Cas9 proteins originating from species other than *Streptococcus pyogenes*. Recognizing that SpRY-ABE8.8/PAH4 is unlikely to be the optimal adenine base editor for correction of the *PAH* c.1222C>T variant, we did not perform an exhaustive analysis of its off-target editing. We also did not evaluate for the possibility of gRNA-independent off-target editing by SpRY-ABE8.8, wherein the deaminase domain of the base editor can spuriously deaminate genomic nucleic acid sequences independently of the Cas9 protein; notably, prior studies have suggested that this phenomenon is minimized when eighth-generation adenine base editors are delivered as mRNAs.^15^^,^^22^ When SpRY-ABE8.8/PAH4 base editing for correction of the *PAH* c.1222C>T variant is compared against a similar base editing approach for correction of the *PAH* c.842C>T (p.Pro281Leu) variant,^9^ also a frequent (albeit much less frequent) PKU variant, the former had substantially less potency in HuH-7 cells (EC_50_ of 750 fmol/cell versus EC_50_ of 64 fmol/cell), less editing activity in the mouse liver when delivered with the same LNP formulation at the same 2.5-mg/kg dose (mean desired on-target editing of 26% versus 39%), and more unwanted bystander editing *in vivo* (2.8% versus 0.8%). Finally, we did not perform a long-term mouse study, although the precedent of the LNP treatment for the *PAH* c.842C>T (p.Pro281Leu) variant resulting in durable normalization of blood Phe levels in PKU mice, through 6 months of observation,^9^ suggests that SpRY-ABE8.8/PAH4 LNP treatment would be similarly durable.

In conclusion, we demonstrate that a base editing strategy is effective in treating a humanized mouse model of the most common pathogenic variant found in individuals with the most common inborn error of metabolism. Future studies will focus on optimizing the base editing approach further and assessing its relative merits and demerits compared with a prime editing approach.

## Data and code availability

The accession number for the next-generation sequencing data reported in this paper is Sequence Read Archive: PRJNA1026949. Other data are available in the Supplemental Data.

## Supplemental information

Document S1. Figures S1–S5 and Tables S1–S3

Document S2. Article plus supplemental information

## References

[1] Vockley J., Andersson H.C., Antshel K.M., Braverman N.E., Burton B.K., Frazier D.M., Mitchell J., Smith W.E., Thompson B.H., Berry S.A., American College of Medical Genetics and Genomics Therapeutics Committee (2014). Phenylalanine hydroxylase deficiency: diagnosis and management guideline. Genet. Med..

[2] Jurecki E.R., Cederbaum S., Kopesky J., Perry K., Rohr F., Sanchez-Valle A., Viau K.S., Sheinin M.Y., Cohen-Pfeffer J.L. (2017). Adherence to clinic recommendations among patients with phenylketonuria in the United States. Mol. Genet. Metab..

[3] Hillert A., Anikster Y., Belanger-Quintana A., Burlina A., Burton B.K., Carducci C., Chiesa A.E., Christodoulou J., Đorđević M., Desviat L.R. (2020). The genetic landscape and epidemiology of phenylketonuria. Am. J. Hum. Genet..

[4] Brooks D.L., Whittaker M.N., Qu P., Musunuru K., Ahrens-Nicklas R.C., Wang X. (2023). Efficient in vivo prime editing corrects the most frequent phenylketonuria variant, associated with high unmet medical need. Am. J. Hum. Genet..

[5] Leuders S., Wolfgart E., Ott T., du Moulin M., van Teeffelen-Heithoff A., Vogelpohl L., Och U., Marquardt T., Weglage J., Feldmann R., Rutsch F. (2014). Influence of PAH genotype on sapropterin response in PKU: results of a single-center cohort study. JIMD Rep..

[6] Burton B.K., Longo N., Vockley J., Grange D.K., Harding C.O., Decker C., Li M., Lau K., Rosen O., Larimore K. (2020). Pegvaliase for the treatment of phenylketonuria: results of the phase 2 dose-finding studies with long-term follow-up. Mol. Genet. Metab..

[7] Komor A.C., Kim Y.B., Packer M.S., Zuris J.A., Liu D.R. (2016). Programmable editing of a target base in genomic DNA without double-stranded DNA cleavage. Nature.

[8] Gaudelli N.M., Komor A.C., Rees H.A., Packer M.S., Badran A.H., Bryson D.I., Liu D.R. (2017). Programmable base editing of AT to GC in genomic DNA without DNA cleavage. Nature.

[9] Brooks D.L., Carrasco M.J., Qu P., Peranteau W.H., Ahrens-Nicklas R.C., Musunuru K., Alameh M.-G., Wang X. (2023). Rapid and definitive treatment of phenylketonuria in variant-humanized mice with corrective editing. Nat. Commun..

[10] Desviat L.R., Pérez B., Gámez A., Sánchez A., García M.J., Martínez-Pardo M., Marchante C., Bóveda D., Baldellou A., Arena J. (1999). Genetic and phenotypic aspects of phenylalanine hydroxylase deficiency in Spain: molecular survey by regions. Eur. J. Hum. Genet..

[11] Zhu T., Ye J., Han L., Qiu W., Zhang H., Liang L., Gu X. (2017). The predictive value of genetic analyses in the diagnosis of tetrahydrobiopterin (BH4)-responsiveness in Chinese phenylalanine hydroxylase deficiency patients. Sci. Rep..

[12] Chatterjee P., Lee J., Nip L., Koseki S.R.T., Tysinger E., Sontheimer E.J., Jacobson J.M., Jakimo N. (2020). A Cas9 with PAM recognition for adenine dinucleotides. Nat. Commun..

[13] Walton R.T., Christie K.A., Whittaker M.N., Kleinstiver B.P. (2020). Unconstrained genome targeting with near-PAMless engineered CRISPR-Cas9 variants. Science.

[14] Richter M.F., Zhao K.T., Eton E., Lapinaite A., Newby G.A., Thuronyi B.W., Wilson C., Koblan L.W., Zeng J., Bauer D.E. (2020). Phage-assisted evolution of an adenine base editor with improved Cas domain compatibility and activity. Nat. Biotechnol..

[15] Gaudelli N.M., Lam D.K., Rees H.A., Solá-Esteves N.M., Barrera L.A., Born D.A., Edwards A., Gehrke J.M., Lee S.J., Liquori A.J. (2020). Directed evolution of adenine base editors with increased activity and therapeutic application. Nat. Biotechnol..

[16] Ponnienselvan K., Liu P., Nyalile T., Oikemus S., Maitland S.A., Lawson N.D., Luban J., Wolfe S.A. (2023). Reducing the inherent auto-inhibitory interaction within the pegRNA enhances prime editing efficiency. Nucleic Acids Res..

[17] Kingwell K. (2022). Base editors hit the clinic. Nat. Rev. Drug Discov..

[18] Kleinstiver B.P., Prew M.S., Tsai S.Q., Topkar V.V., Nguyen N.T., Zheng Z., Gonzales A.P.W., Li Z., Peterson R.T., Yeh J.R.J. (2015). Engineered CRISPR-Cas9 nucleases with altered PAM specificities. Nature.

[19] Hu J.H., Miller S.M., Geurts M.H., Tang W., Chen L., Sun N., Zeina C.M., Gao X., Rees H.A., Lin Z., Liu D.R. (2018). Evolved Cas9 variants with broad PAM compatibility and high DNA specificity. Nature.

[20] Nishimasu H., Shi X., Ishiguro S., Gao L., Hirano S., Okazaki S., Noda T., Abudayyeh O.O., Gootenberg J.S., Mori H. (2018). Engineered CRISPR-Cas9 nuclease with expanded targeting space. Science.

[21] Miller S.M., Wang T., Randolph P.B., Arbab M., Shen M.W., Huang T.P., Matuszek Z., Newby G.A., Rees H.A., Liu D.R. (2020). Continuous evolution of SpCas9 variants compatible with non-G PAMs. Nat. Biotechnol..

[22] Musunuru K., Chadwick A.C., Mizoguchi T., Garcia S.P., DeNizio J.E., Reiss C.W., Wang K., Iyer S., Dutta C., Clendaniel V. (2021). In vivo CRISPR base editing of PCSK9 durably lowers cholesterol in primates. Nature.

