# Supplementary material for: A base editing strategy using mRNA-LNPs for in vivo correction of the most frequent phenylketonuria variant
Source: HGG Adv. 2023 Nov 2;5(1):100253. doi: 10.1016/j.xhgg.2023.100253 (PMC10800763; doi:10.1016/j.xhgg.2023.100253)
Supplement: Document S1. Figures S1–S5 and Tables S1–S3 [file mmc1.pdf]

HGGA, Volume *5*

Supplemental information

A base editing strategy using mRNA-LNPs for *in vivo* correction of the most frequent phenylketonuria variant

Dominique L. Brooks, Madelynn N. Whittaker, Hooda Said, Garima Dwivedi, Ping Qu, Kiran Musunuru, Rebecca C. Ahrens-Nicklas, Mohamad-Gabriel Alameh, and Xiao Wang

Figure S1. On-target and Bystander Editing with Plasmid Transfections, Related to Figure 1B.

Results of next-generation sequencing, with the mean percentage for each nucleotide at each position indicated. Values ≤ 0.1% are not shown. Green = A, yellow = G, orange = C, purple = T. The red box indicates the c.1222C>T adenine; black boxes indicate sites of unwanted adenine bystander editing.

Reference	A	A	G	G	G	C	C	A	A	G	G	T	A	T	T	G	T	G	G	C	A	G	C
sgRNA																							
SpG-ABE7.10/PAH1	99.9	99.9						98.1	99.3				99.8								99.9		
			100	99.9	100			1.9	0.6	100	100		0.2			99.9		100	99.9			99.9	
						99.9	99.9													99.9			100
												99.9		100	99.9		99.9						
SpG-ABE8.20/PAH1	99.4	94.1						92.6	99				98.8								99.9		
	0.6	5.8	100	100	100	0.5	0.2	7.4	0.9	100	100		1.2			100		100	99.9			99.9	
						98.4	99.7													99.9			100
						1.0						99.8		100	99.9		99.9						
SpG-ABE8e/PAH1	99.6	97.9						92.4	99				98.9								99.9		
	0.3	2.1	100	99.9	100			7.6	0.9	100	100		1.1			100		99.9	99.9			99.9	
						99.6	99.9													99.9			100
						0.2						99.9		99.9	100		99.9						
SpRY-ABE8e/PAH1	99.8	98.3						95	99.1				98.7								99.9		
	0.2	1.6	100	100	100			5.0	0.8	100	100		1.3			100		100	100			99.9	
						99.6	99.8													99.9			100
						0.3						99.9		99.9	99.9		99.9						
SpRY-ABE8.8/PAH1	99.9	99.7						98.3	99.6				99.7								99.9		
		0.2	100	99.9	99.9			1.6	0.4	100	100		0.2			100		100	99.9			99.9	
						99.9	100													99.9			100
												99.9		99.9	99.9		99.9						
SpRY-ABE8e/PAH2	99.8	99.9						94.2	96.6				98.4								99.9		
			100	100	99.9		0.2	5.7	3.3	100	100		1.5			100		100	100			99.9	
						99.8	99.7													99.9			100
						0.2						99.9		100	99.9		99.9						
SpRY-ABE8.8/PAH2	100	99.9						96.7	99.1				99.7								99.9		
			100	99.9	100			3.3	0.8	100	100		0.3			100		100	100			100	
						99.9	99.9													99.9			100
												99.9		100	100		99.9						
iSpyMac-ABE8.8/PAH3	99.9	99.9						90.8	97.3				99.7								99.9		
			100	99.9	100			9.1	2.6	100	100		0.3			100		100	99.9			99.9	
						99.9	99.9													99.9			100
												99.9		100	100		99.9						
SpRY-ABE8e/PAH3	99.9	99.9						94.1	95.9				98.5								99.9		
			99.9	100	99.9		0.2	5.9	4.1	100	100		1.4			100		99.9	100			99.9	
						99.8	99.6													99.9			100
												99.9		100	100		99.9						
SpRY-ABE8.8/PAH3	99.9	99.9						95.7	98.5				99.8								99.9		
			100	99.9	99.9			4.2	1.4	100	100		0.2			100		100	99.9			99.9	
						99.8	99.9													99.9			100
												99.9		100	100		99.9						
iSpyMac-ABE8.8/PAH4	99.9	99.9						93.3	96.8				99.2								99.9		
			100	99.9	99.9			6.6	3.1	100	100		0.8			100		99.9	100			99.9	
						99.9	99.9													99.9			100
												99.9		100	99.9		99.9						
SpRY-ABE8e/PAH4	99.9	99.9					0.2	93.7	94.4				96.6								99.9		
			100	99.9	100		0.2	6.3	5.6	100	100		3.4			100		99.9	99.9			99.9	
						99.9	99.6													99.9			100
												99.9		99.9	99.9		99.9						
SpRY-ABE8.8/PAH4	100	99.9						86.7	93.6				99								99.9		
			100	99.9	100			13.3	6.3	100	100		0.9			100		99.9	99.9			100	
						99.8	99.7													99.9			100
												99.9		100	99.9		99.9						
SpRY-ABE8e/PAH5	99.9	99.9						90.7	91				91.9								99.7		
			100	99.9	99.9			9.2	8.9	100	100		8.1			100		100	100		0.2	99.9	
						99.9	99.9													99.9			100
												99.9		100	99.9		100						
SpRY-ABE8.8/PAH5	99.9	99.9						90.3	91.7				96.7								99.9		
			99.9	99.9	99.9			9.7	8.2	100	100		3.2			100		99.9	99.9			99.9	
						99.9	99.9													99.9			100
												99.9		99.9	99.9		99.9						
ABE8.8/PAH5	99.9	99.9						99.0	99.5				99.7								99.9		
			100	99.9	99.9			1.0	0.4	100	100		0.3			100		100	100			100	
						99.9	100													99.9			100
												99.9		99.9	100		99.9						
SpG-ABE7.10/PAH6	99.9	99.9						98.8	98.7				93.1								99.9		
			100	99.9	100			1.2	1.3	100	100		6.9			100		100	99.9			99.9	
						99.9	100													100			100
												99.9		100	99.9		99.9						
SpG-ABE8.20/PAH6	99.9	99.9						83.2	84.2				80.9								99.8		
			100	99.9	99.9			16.7	15.8	100	100		19.1			100		100	99.9			99.9	
						99.9	99.9													99.9			100
												99.8		99.9	100		99.9						
SpG-ABE8e/PAH6	99.9	99.9						94.3	94.3				94.1								99.7		
			99.9	99.9	100			5.6	5.6	100	100		5.9			100		99.9	99.9		0.3	100	
						99.9	99.9													99.9			100
												99.9		99.9	99.9		99.8						
SpRY-ABE8e/PAH6	99.9	99.9						93.6	93.7				93.3								99.6		
			100	99.9	100			6.4	6.3	100	100		6.7			100		100	99.9		0.4	100	
						99.9	100													99.9			100
												99.9		99.9	99.9		99.9						
SpRY-ABE8.8/PAH6	99.9	99.9						95.2	93.7				90.3								99.9		
			100	99.9	100			4.7	6.2	100	100		9.6			100		100	100			100	
						99.9	100													99.9			100
												99.9		99.9	99.9		99.9						

Figure S2. On-target and Bystander Editing with LNP Transfections, Related to Figure 1C.

Results of next-generation sequencing, with the mean percentage for each nucleotide at each position indicated. Values ≤ 0.1% are not shown. Green = A, yellow = G, orange = C, purple = T. The red box indicates the c.1222C>T adenine; black boxes indicate sites of unwanted adenine bystander editing.

Reference		A	A	G	G	G	C	C	A	A	G	G	T	A	T	T	G	T	G	G	C	A	G	C
sgRNA																								
39 fg/cell SpRY-ABE8.8/PAH4	A	99.9	99.9						99.2	99.8				99.8								99.9		
	G			100	99.9	99.9			0.8	0.2	100	100					99.9		100	100			100	
	C						99.9	99.9													100			100
	T	99.9	99.9						99.2	99.8				99.8								99.9		
78 fg/cell SpRY-ABE8.8/PAH4	A	99.9	99.9						98.5	99.5				99.8								99.9		
	G			99.9	99.9	99.9			1.5	0.4	100	99.9		0.2			100		100	100			99.9	
	C						99.9	100													100			100
	T												99.8		100	100		99.9						
156 fg/cell SpRY-ABE8.8/PAH4	A	99.9	99.9						94.3	98.7				99.4								99.9		
	G			99.9	99.9	99.9			5.7	1.2	100	100		0.6			99.9		100	100			100	
	C						99.9	100													100			100
	T												99.8		100	100		99.9						
313 fg/cell SpRY-ABE8.8/PAH4	A	99.9	99.9						86	96.6				98.5								99.9		
	G			100	99.9	99.9			13.9	3.3	99.9	100		1.5			99.9		100	99.9			99.9	
	C						99.9	100													100			100
	T												99.8		99.9	99.9		99.9						
625 fg/cell SpRY-ABE8.8/PAH4	A	99.9	99.8						69.3	92.4				97.0								99.8		
	G			100	99.9	99.9			30.7	7.5	99.9	100		3.0			100		99.9	99.9			99.9	
	C						99.9	99.9													99.9			99.9
	T												99.8		100	100		99.9						
1250 fg/cell SpRY-ABE8.8/PAH4	A	99.9	99.9						49.1	86.5				93.8								99.9		
	G			99.9	99.9	99.9			50.9	13.4	99.9	100		6.2			99.9		99.9	99.9			99.9	
	C						99.8	99.9													99.9			99.9
	T												99.7		100	100		99.8						
2500 fg/cell SpRY-ABE8.8/PAH4	A	99.9	99.9					0.3	26.6	77.3				88.5								99.8		
	G			99.9	99.9	99.9			73.4	22.6	99.9	100		11.5			99.9		100	100			99.9	
	C						99.9	99.6													99.8			100
	T												99.7		100	99.9		99.8						
5000 fg/cell SpRY-ABE8.8/PAH4	A	99.9	99.9						27.7	78.8				90								99.9		
	G			100	99.9	99.9			72.2	21.1	99.9	100		9.9			99.9		99.9	99.9			99.9	
	C						99.9	99.9													99.9			100
	T												99.8		100	100		99.9						
10000 fg/cell SpRY-ABE8.8/PAH4	A	99.9	100				0.2		28.5	79			0.2	91.4								100		
	G			99.9	99.9	99.9			71.4	20.7	100	100		8.6			100		99.9	99.9			100	
	C						99.8	99.8					0.2								99.9			100
	T									0.2			99.4		100	99.9		99.9						

Figure S3. Phenylketonuria Phenotypes in Humanized R408W Mice.

(A) Sanger sequencing chromatograms showing the generation of a humanized mouse model via Cas9-mediated homology-directed repair in mouse zygotes. At the top is sequence from a wild-type C57BL/6J mouse. At the bottom is sequence from a mouse homozygous for the humanized *Pah* c.1222C>T allele. The red arrow indicates the site of the c.1222C>T variant, and the black arrows indicate the sites of additional changes that humanize the local region of the mouse *Pah* gene.
(B) Homozygous R408W mice with PKU as evidenced by mild hypopigmentation of the fur, on left, compared to heterozygous R408W non-PKU littermates, on right.
(C) Blood phenylalanine levels and weights of homozygous R408W PKU (*n* = 5 animals) and heterozygous R408W non-PKU (*n* = 3 animals) age-matched (6 weeks of age) littermates. Lines = mean values. *P* values calculated with unpaired t-test.

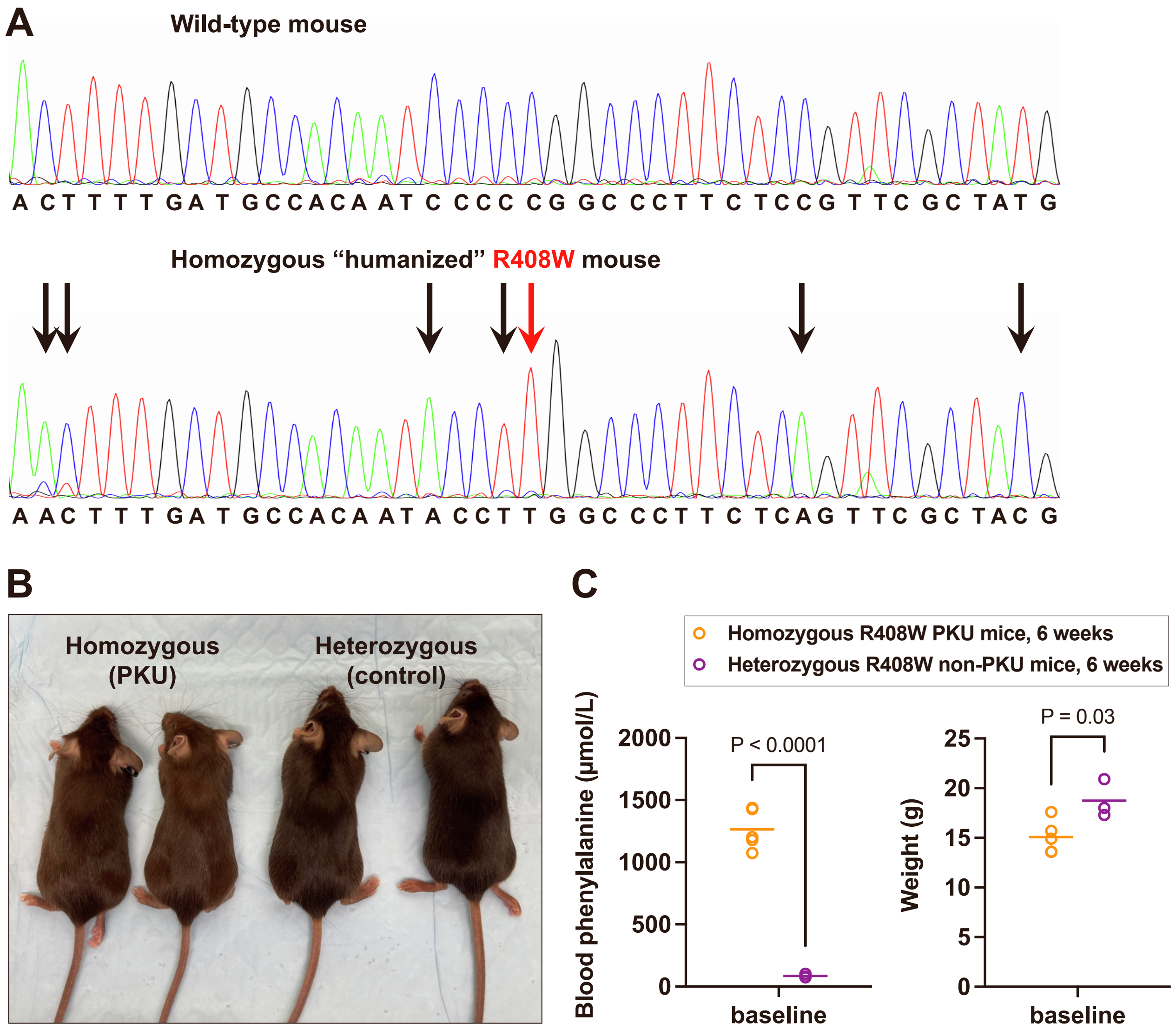

Figure S4. Assessment of Mouse Liver Following LNP Treatment or Vehicle Treatment.

(A) Blood aspartate aminotransferase (AST) levels in homozygous PKU mice following treatment with 5 mg/kg dose of SpRY-ABE8.8/PAH4 LNPs (*n* = 3 animals) or with 2.5 mg/kg dose of LNPs (*n* = 3 animals), comparing levels at various timepoints up to 7 days following treatment to levels in vehicle-treated homozygous PKU control (*n* = 4 animals) and vehicle-treated heterozygous non-PKU control (*n* = 4 animals) age-matched (approximately 8 weeks of age) colonymates (1 blood sample per timepoint).
(B) Blood alanine aminotransferase (ALT) levels in the LNP-treated or vehicle-treated PKU or non-PKU mice shown in (A).
(C) Representative liver histology (hematoxylin/eosin staining) at 20× magnification upon necropsy at 1 week after LNP treatment of PKU mice or vehicle treatment of a non-PKU mouse shown in (A) and (B).
Lines in pictures = distance of 50 μm.
Lines in graphs = mean values.

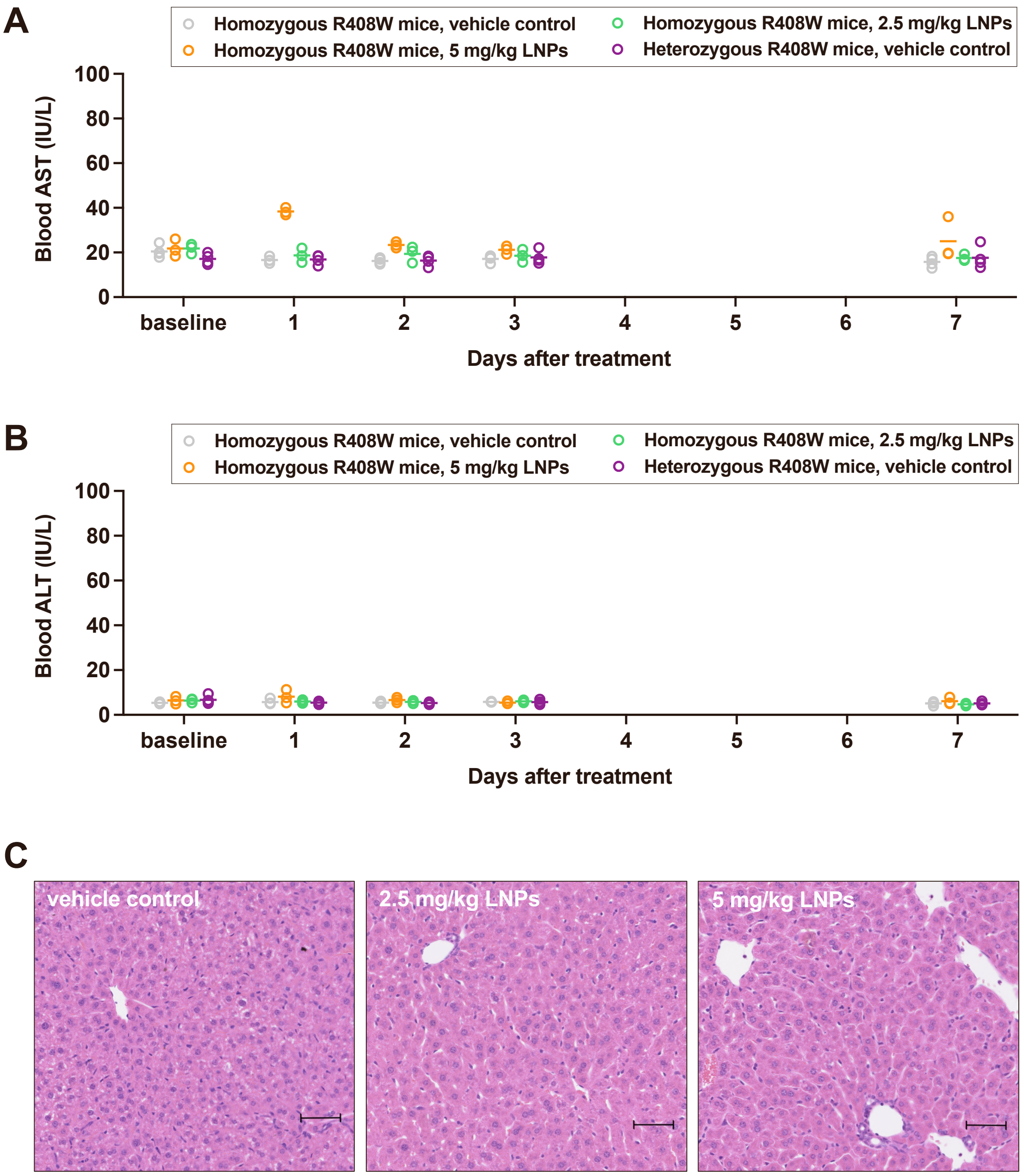

Figure S5. Prime Editing to Correct *PAH* c.1222C>T Variant in Humanized Mice.

(A) Blood phenylalanine levels in homozygous PKU mice following treatment with PEmax/P56/N19 LNPs (n = 3 animals) at various timepoints up to 4 days following treatment (1 blood sample per timepoint). Lines = mean values.
(B) Corrective *PAH* c.1222C>T editing (determined from genomic DNA) in each of 8 liver samples (two samples each from the four lobes) collected from each treated mouse upon necropsy 1 week after treatment, calculated as the proportion of aligned sequencing reads in which the c.1222C>T adenine variant is edited to guanine, irrespective of other edits.

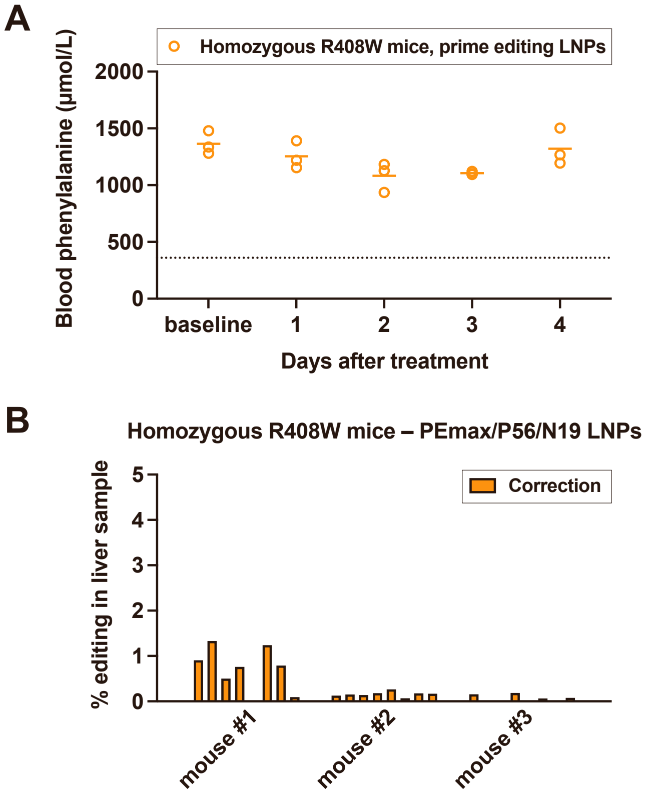

Table S1. Cytokine and Chemokine Levels in R408W Humanized Mice Following LNP Treatment or Vehicle Treatment.

Time	IFN-γ	CXCL-1	TNF-α	MCP-1	IL-12p70	RANTES	IL-1β	IP-10	GM-CSF	IL-10	IFN-β	IFN-α	IL-6
**Homozygous R408W PKU mouse, 8 weeks of age, 2.5 mg/kg SpRY-ABE8.8/PAH4 LNPs**													
**baseline**	3.40	29.17	9.40	11.59	1.52	33.83	1.43	231.12	4.35	11.01	0.00	3.41	7.86
**4 hours**	4.45	109.57	17.59	115.68	1.73	31.87	13.04	2374.70	2.60	3.49	0.00	10.27	1675.05
**24 hours**	3.40	24.64	6.06	7.73	1.62	32.43	1.25	296.92	1.64	0.44	0.00	3.41	32.90
**Homozygous R408W PKU mouse, 8 weeks of age, vehicle control**													
**baseline**	2.20	15.97	7.69	4.97	1.20	30.19	2.72	135.86	2.50	0.73	0.00	2.50	5.75
**4 hours**	2.65	18.12	5.04	13.43	1.66	24.09	3.73	90.19	1.34	0.44	0.00	2.23	289.85
**24 hours**	1.59	25.62	8.25	6.75	1.26	16.01	6.62	128.72	0.93	1.46	0.00	1.39	298.85
**Heterozygous R408W non-PKU mouse, 8 weeks of age, vehicle control**													
**baseline**	1.89	8.55	4.08	9.58	1.14	20.81	0.29	66.45	3.49	4.74	0.00	1.73	2.12
**4 hours**	2.35	43.47	5.04	16.40	1.87	21.90	4.55	109.61	2.14	3.49	0.00	2.50	198.09
**24 hours**	1.89	27.58	6.06	8.76	1.35	27.40	8.55	142.82	2.88	4.74	0.00	2.67	67.20

All cytokine/chemokine measurements are in units of pg/mL.

Table S2. Assessment of Off-target Editing in *PAH* c.1222C>T Homozygous HuH-7 Cells.

Genomic site	Protospacer/PAM sequence[a]	Amplicon (chromosome: position range)[b]	Treated sample 1[c]	Treated sample 2	Treated sample 3	Control sample 1	Control sample 2	Control sample 3
PAH	GGCCAAGGTATTGTGGCAGCAAA	chr12:102840425+102840539	75.87	78.73	77.76	0.20	0.16	0.20
OT1	GGCCAAGGgATTGTGG-AGCTGT	chr1:67956357-67956526	0.08	0.06	0.09	0.10	0.06	0.07
OT2	GGCCAAGcTATTGCTGGCAGCCAG	chr1:165405262+165405443	0.06	0.06	0.04	0.04	0.04	0.03
OT3	GGCCAAGGT-TTcTGGCAGCTGG	chr1:182646888+182647080	0.06	0.06	0.08	0.04	0.04	0.03
OT4	GcCCAAGGTATTGTGGCA-CCGC	chr12:98762684-98762861	0.07	0.07	0.07	0.08	0.06	0.05
OT5	tGCCAAGGTATTGT-GCAGCCAG	chr13:56216949+56217104	0.09	0.05	0.13	0.13	0.06	0.04
OT6	GGCCAAGG-AaTGTGGCAGCTTC	chr13:102535487-102535594	0.11	0.08	0.10	0.09	0.11	0.08
OT7	GGCC-AGGcATTGTGGCAGCACC	chr13:112399414-112399538	0.06	0.06	0.04	0.04	0.09	0.06
OT8	GGCCAAGGT-TTGTGGCAaCCCT	chr14:98620044+98620168	0.05	0.07	0.04	0.02	0.04	0.04
OT9	GG-CAAGGTgTTGTGGCAGCTTT	chr17:79400839-79400958	0.03	0.03	0.03	0.03	0.05	0.04
OT10	GGCCAAGG-ATTGTGtCAGCCCA	chr5:156096522-156096673	0.04	0.09	0.06	0.05	0.08	0.06
OT11	GGCC-AGGTtTTGTGGCAGCATC	chr5:168457657-168457813	0.03	0.04	0.04	0.04	n.d.	0.03
OT12	aGCCAAGG-ATTGTGGCAGCTAC	chrX:105510661+105510860	0.18	0.20	0.11	0.06	0.06	0.05
OT13	GGCCAAGGTA--GTGGCAGaGCA	chr1:39317216+39317406	0.06	0.05	0.06	0.06	0.03	0.05
OT14	GGCCA--GTATgGTGGCAGCAGA	chr1:91658136-91658280	0.05	0.06	0.02	0.05	0.04	0.05
OT15	GGCCAAGGTAT--TGGCAGgTAT	chr1:143710105-143710246	0.09	0.09	0.08	0.08	0.07	0.11
OT16	GtCCAAGGTA--GTGGCAGCAAA	chr1:184534886+184535019	0.06	0.05	0.05	0.07	0.08	0.04
OT17	GGCCAAGGTg--GTGGCAGCCAC	chr11:16790220+16790462	0.06	0.01	0.03	0.02	0.04	0.04
OT18	GG--AAGaTATTGTGGCAGCCGT	chr11:42929612-42929802	0.09	0.12	0.07	0.08	0.07	0.09
OT19	GG--AAGGTATTGTtGCAGCTGT	chr11:43718128+43718289	0.06	0.11	0.10	0.07	0.11	0.07
OT20	GcCCAAGGTATTGTGGCACCGCAGA	chr12:98762684-98762861	0.09	0.04	0.07	0.07	0.03	0.05
OT21	GGC--AGGTATTGTtGCAGCCCC	chr13:95505215+95505411	0.10	0.04	0.04	0.07	0.07	0.04
OT22	GGCCAAG--ATTGTGtCAGCTAG	chr15:73613514+73613688	0.07	0.05	0.09	0.06	0.05	0.09
OT23	GG--AAGGTATTcTGGCAGCTCT	chr16:1606113-1606257	0.02	0.09	0.04	0.06	0.04	0.07
OT24	GGCCAAaGTATTGTGG--GCTAA	chr16:65767602-65767791	0.04	0.05	0.03	0.05	0.05	0.05
OT25	GG--gAGGTATTGTGGCAGCTCC	chr17:50567554-50567721	n.d.	n.d.	n.d.	n.d.	n.d.	n.d.
OT26	GGCCAAGGTAgTGTG--AGCTGA	chr18:36992979+36993082	n.d.	n.d.	n.d.	n.d.	n.d.	n.d.
OT27	GGCCAgG--ATTGTGGCAGCAGA	chr2:21132399+21132532	0.13	0.07	0.05	0.04	0.06	0.04
OT28	G--CAAGGTATTGTGGCtGCCAC	chr2:65797812+65797991	0.07	0.07	n.d.	0.06	0.06	0.04
OT29	GGCCAAGG--TTGTaGCAGCTTG	chr2:74493829-74493998	0.02	0.04	0.03	0.03	0.03	0.05
OT30	GGCCAA--TATTGaGGCAGCTAT	chr2:75098514-75098650	0.05	0.07	0.06	0.03	0.07	0.04
OT31	GGCCAAGGTAgTGTGG--GCAGA	chr2:170268241+170268437	0.10	0.09	0.10	0.07	0.09	0.10
OT32	GGCCACCAGGaATTGTGGCAGCCAA	chr20:49750986+49751159	0.03	0.03	0.02	0.02	0.05	0.04
OT33	GGCCAAGG--cTGTGGCAGCTTC	chr20:57441083-57441206	0.01	0.03	0.01	0.01	0.01	0.02
OT34	GGCCAAGGTtTTGT--CAGCCAC	chr3:3727491-3727618	0.05	0.06	0.06	0.04	0.03	0.05
OT35	GGCCAAGaTATTGTGGC--CAAT	chr4:33129101-33129296	0.09	n.d.	0.09	0.08	0.07	0.11
OT36	GGCCAAGGgATTGTG--AGCAGA	chr4:176650261-176650399	0.08	0.07	0.04	0.04	0.06	0.06
OT37	GcCCAAGG--TTGTGGCAGCCCA	chr5:15842116+15842313	n.d.	n.d.	n.d.	n.d.	n.d.	n.d.
OT38	GtCCAAGGTATTGT--CAGCTTG	chr5:42644125-42644285	0.07	0.06	0.07	0.12	0.09	0.06
OT39	GGCCAAGGTgT--TGGCAGCTGC	chr5:138315728+138315864	0.05	0.03	0.05	0.03	0.07	0.04
OT40	GGCCAAGGTATTGTG--AGgGGA	chr6:23532313+23532467	0.07	0.06	0.05	0.10	0.07	0.08
OT41	GG--AAtGTATTGTGGCAGCTCC	chr6:36145377-36145550	0.06	0.06	0.07	0.05	0.07	0.05
OT42	GGCCAAGGTA--GTGGCAaCAGA	chr6:50411635+50411817	0.08	0.05	0.08	0.06	0.09	0.08
OT43	GtCCAAGGTAT--TGGCAGCTTC	chr6:68856951+68857064	0.06	0.08	0.06	0.08	0.08	0.07
OT44	GGCCAAGGTAT--TGGCAGgGAT	chr6:115167457+115167613	0.07	0.04	0.08	0.11	0.08	0.05
OT45	GGCCtAGGTATTGT--CAGCTCT	chr6:123648344+123648515	n.d.	n.d.	n.d.	n.d.	n.d.	n.d.
OT46	GGCCAAGGTA--GTGGgAGCTTT	chr7:11182970-11183157	0.07	0.09	0.06	0.09	0.05	0.07
OT47	GGCCAAGGTA--GTGGCAGtAAG	chr8:10159022+10159160	0.05	0.08	0.08	0.10	0.07	0.06
OT48	GG--AAGGTATTaTGGCAGCAGC	chr8:80885442+80885634	0.07	0.06	0.08	0.07	0.10	0.07
OT49	GGC--AGGTATTGTGGCAGaAGT	chr8:122080739-122080887	0.07	0.05	0.06	0.06	0.05	0.05
OT50	GcCCAAGGTAT--TGGCAGCTTT	chrX:146875655+146875796	n.d.	n.d.	n.d.	n.d.	n.d.	n.d.
OT51	aGCCAcGGTATTGTGGCAGCTTG	chr1:160709019+160709185	0.03	n.d.	0.03	0.06	0.02	0.07
OT52	GGaCAAGGTATTGTGGgAGCCCA	chr10:58773438+58773613	0.05	n.d.	0.09	0.08	0.11	n.d.
OT53	GGCCAAGGTAcTGTGtCAGCCAG	chr12:51752733-51752923	0.10	n.d.	0.07	0.08	0.13	0.14
OT54	GGCCAAGGaATTcTGGCAGCCAC	chr14:78442315+78442449	0.06	n.d.	0.08	0.07	0.10	0.09
OT55	GGCCAAGGTgaTGTGGCAGCATG	chr15:62831968-62832156	0.07	n.d.	0.10	0.06	0.07	0.09
OT56	GGCCAAGGTATgGTGcCAGCTTG	chr2:118901813-118901955	0.04	0.06	0.05	0.08	0.05	0.08
OT57	GGCCAAGGgtTTGTGGCAGCACA	chr8:47691583-47691764	0.08	0.04	0.08	0.05	0.06	0.07

[a]The *PAH* on-target sequence and top in-silico-nominated candidate off-target sequences with mismatched positions and bulges indicated by lower case letters and dashes.

[b]Coordinates in the GRCh38/hg38 assembly of the human genome; "+" and "-" indicate forward or reverse orientation of protospacer/PAM sequence.

[c]n.d. = PCR was unsuccessful for the genomic site.

Table S3. PCR Primers for Next-generation Sequencing.

Site	Forward primer	Reverse primer
PAH (human)	TCGTCGGCAGCGTCAGATGTGTATAAGAGACAG TGTCCAAGACCTCAATCCTTTG	GTCTCGTGGGCTCGGAGATGTGTATAAGAGACAG CACTCAAGCCTGTGGTTTTG
PAH (mouse)	TCGTCGGCAGCGTCAGATGTGTATAAGAGACAG GGCTCACAGCAAGGAAAAAG	GTCTCGTGGGCTCGGAGATGTGTATAAGAGACAG AGGTAGTCCCAGCAGGTGTG
PAH (OT analysis)	TCGTCGGCAGCGTCAGATGTGTATAAGAGACAG AAGCTGCTGGGTATTGTCCA	GTCTCGTGGGCTCGGAGATGTGTATAAGAGACAG TCAAGCCTGTGGTTTTGGTC
OT1	TCGTCGGCAGCGTCAGATGTGTATAAGAGACAG GGTTTCAAATCAGTCTGGCCA	GTCTCGTGGGCTCGGAGATGTGTATAAGAGACAG CCTGCCCTTCAAAACTAGTGG
OT2	TCGTCGGCAGCGTCAGATGTGTATAAGAGACAG TCCAACTCAGGGCTTCAATTAA	GTCTCGTGGGCTCGGAGATGTGTATAAGAGACAG TGTATCCTGTTGTATGCCGAAG
OT3	TCGTCGGCAGCGTCAGATGTGTATAAGAGACAG GGCTTCTCGGGTCTGGAA	GTCTCGTGGGCTCGGAGATGTGTATAAGAGACAG TGCTGTGATCAAAAGGGATGA
OT4	TCGTCGGCAGCGTCAGATGTGTATAAGAGACAG GAGATGCAAAGCCCAGTGAG	GTCTCGTGGGCTCGGAGATGTGTATAAGAGACAG TCCTTTCCTGTCCTCCCAAC
OT5	TCGTCGGCAGCGTCAGATGTGTATAAGAGACAG CGACTTTGCACCTTCCTG	GTCTCGTGGGCTCGGAGATGTGTATAAGAGACAG AAGTGGACTCTCATGCAGGT
OT6	TCGTCGGCAGCGTCAGATGTGTATAAGAGACAG GGGGATGTGGCAGTAGAAGT	GTCTCGTGGGCTCGGAGATGTGTATAAGAGACAG TCTCCCTTTCCCAGCTTCTG
OT7	TCGTCGGCAGCGTCAGATGTGTATAAGAGACAG CTGGCTCTCTCTGCACCTG	GTCTCGTGGGCTCGGAGATGTGTATAAGAGACAG GAAATGTCTCATGGGCAGGC
OT8	TCGTCGGCAGCGTCAGATGTGTATAAGAGACAG GGCACTAATCCATTCATGAAGGT	GTCTCGTGGGCTCGGAGATGTGTATAAGAGACAG TGGCCCAGATAGAGTTGCTC
OT9	TCGTCGGCAGCGTCAGATGTGTATAAGAGACAG CACAGGGATGCTAGCTAGGC	GTCTCGTGGGCTCGGAGATGTGTATAAGAGACAG CAAAACCGAAGTCCCCACAC
OT10	TCGTCGGCAGCGTCAGATGTGTATAAGAGACAG TGCTTGCTTAGAAAATCCAGGG	GTCTCGTGGGCTCGGAGATGTGTATAAGAGACAG TCATTGCCCCATCTGGTGAA
OT11	TCGTCGGCAGCGTCAGATGTGTATAAGAGACAG CAGTGACCAGAGCTTCCATG	GTCTCGTGGGCTCGGAGATGTGTATAAGAGACAG CCACAGCCAAAATCACACCA
OT12	TCGTCGGCAGCGTCAGATGTGTATAAGAGACAG AGGCAGAAGAATAGGTCAGAGT	GTCTCGTGGGCTCGGAGATGTGTATAAGAGACAG CTGGGGCGAAAATCAAGTGT
OT13	TCGTCGGCAGCGTCAGATGTGTATAAGAGACAG CTGGCTTAGTGATGTGAAGGAC	GTCTCGTGGGCTCGGAGATGTGTATAAGAGACAG GAATCTCGAGCTGGGCAATG
OT14	TCGTCGGCAGCGTCAGATGTGTATAAGAGACAG AGGATCACTCTGGCTACTATGT	GTCTCGTGGGCTCGGAGATGTGTATAAGAGACAG TCCTTTGAATCTGACCCTTTCTT
OT15	TCGTCGGCAGCGTCAGATGTGTATAAGAGACAG TGCGTCAGGACAGAAAGTTTG	GTCTCGTGGGCTCGGAGATGTGTATAAGAGACAG TGCTACATAAAACGAAACCACCA
OT16	TCGTCGGCAGCGTCAGATGTGTATAAGAGACAG GGCAGAGAGTTCAGAAGGGT	GTCTCGTGGGCTCGGAGATGTGTATAAGAGACAG AGCACCCAAAGACCTCAAGT
OT17	TCGTCGGCAGCGTCAGATGTGTATAAGAGACAG AGAAGGACACTCAACTGGCA	GTCTCGTGGGCTCGGAGATGTGTATAAGAGACAG GAAGACAATCCCAGTGCTCT
OT18	TCGTCGGCAGCGTCAGATGTGTATAAGAGACAG GGGAACTTCACCACTGTTGAG	GTCTCGTGGGCTCGGAGATGTGTATAAGAGACAG GCTCTGCTTTTCCCTCCAG
OT19	TCGTCGGCAGCGTCAGATGTGTATAAGAGACAG AAAGCAAGCCACAGGACCAG	GTCTCGTGGGCTCGGAGATGTGTATAAGAGACAG TGATATGTTCAGAGGGCAGACT
OT20	TCGTCGGCAGCGTCAGATGTGTATAAGAGACAG GAGATGCAAAGCCCAGTGAG	GTCTCGTGGGCTCGGAGATGTGTATAAGAGACAG TCCTTTCCTGTCCTCCCAAC
OT21	TCGTCGGCAGCGTCAGATGTGTATAAGAGACAG AGGTTCTGAGCGTCCACA	GTCTCGTGGGCTCGGAGATGTGTATAAGAGACAG CCCCATGTGCCCATTGTAAG
OT22	TCGTCGGCAGCGTCAGATGTGTATAAGAGACAG GTTCTCCACTTAGTAATTTCAGA	GTCTCGTGGGCTCGGAGATGTGTATAAGAGACAG GTCCAGCATTTTGTTACCGC
OT23	TCGTCGGCAGCGTCAGATGTGTATAAGAGACAG ATCTTCCTCTGTCAGTGCCC	GTCTCGTGGGCTCGGAGATGTGTATAAGAGACAG CCCTGCTGACACCTGATTTT
OT24	TCGTCGGCAGCGTCAGATGTGTATAAGAGACAG CCCCACATGCTCCATCAGAA	GTCTCGTGGGCTCGGAGATGTGTATAAGAGACAG TGGGGCAAAAGCGATCATTT
OT25	TCGTCGGCAGCGTCAGATGTGTATAAGAGACAG GAGAGCAGGAGATTGGGGAA	GTCTCGTGGGCTCGGAGATGTGTATAAGAGACAG AGCAAGATTCCACCTGCTCT
OT26	TCGTCGGCAGCGTCAGATGTGTATAAGAGACAG CACCTGTAGTCCCAGCTCC	GTCTCGTGGGCTCGGAGATGTGTATAAGAGACAG CCAGGTTGAAGTGCAGTGTT

OT27	TCGTCGGCAGCGTCAGATGTGTATAAGAGACAG GATGGGGTGGAGTTAAGGCT	GTCTCGTGGGCTCGGAGATGTGTATAAGAGACAG TGGGTTCATCTCCAGAGCAT
OT28	TCGTCGGCAGCGTCAGATGTGTATAAGAGACAG TTAGGATCTCAGTGGGTGGG	GTCTCGTGGGCTCGGAGATGTGTATAAGAGACAG AGAGCAACTTTCATCCCTGC
OT29	TCGTCGGCAGCGTCAGATGTGTATAAGAGACAG ACATGGGGAAAGGCTAATTACC	GTCTCGTGGGCTCGGAGATGTGTATAAGAGACAG AAGTCCCTTACCAGCTCCTG
OT30	TCGTCGGCAGCGTCAGATGTGTATAAGAGACAG TAGGGATGTGGGTTTCAGGG	GTCTCGTGGGCTCGGAGATGTGTATAAGAGACAG TGAGAAAGATGAAGAGAGACCT
OT31	TCGTCGGCAGCGTCAGATGTGTATAAGAGACAG TCTGGAGAAAAGGGCAAACTC	GTCTCGTGGGCTCGGAGATGTGTATAAGAGACAG CACTCTGGCCTATAGATTTTACA
OT32	TCGTCGGCAGCGTCAGATGTGTATAAGAGACAG ACATGAAACACAAGGGCAGG	GTCTCGTGGGCTCGGAGATGTGTATAAGAGACAG ATGGGCTCAGGGAAGAAGAC
OT33	TCGTCGGCAGCGTCAGATGTGTATAAGAGACAG CATGCCAGGCCTCTCACC	GTCTCGTGGGCTCGGAGATGTGTATAAGAGACAG GAATCCCTGTCCCACCCTG
OT34	TCGTCGGCAGCGTCAGATGTGTATAAGAGACAG GTGGTGCCAGAGGTAAGACT	GTCTCGTGGGCTCGGAGATGTGTATAAGAGACAG TCACCTAAACACGATGCCCT
OT35	TCGTCGGCAGCGTCAGATGTGTATAAGAGACAG GTAGATACTACTAGGGCTCACT	GTCTCGTGGGCTCGGAGATGTGTATAAGAGACAG CAGACTGGCAGGAGAAGAGA
OT36	TCGTCGGCAGCGTCAGATGTGTATAAGAGACAG CTGGGCACATGGGAATGATG	GTCTCGTGGGCTCGGAGATGTGTATAAGAGACAG GGAATTGCACAGTGACCCTT
OT37	TCGTCGGCAGCGTCAGATGTGTATAAGAGACAG CAGACACTCAACGCCATCAG	GTCTCGTGGGCTCGGAGATGTGTATAAGAGACAG CCCATGCAAGTCTGATGTCC
OT38	TCGTCGGCAGCGTCAGATGTGTATAAGAGACAG TGGGTGGTGCTGTGAACTAT	GTCTCGTGGGCTCGGAGATGTGTATAAGAGACAG AGGTATGCTGTCTTCTAGGTGA
OT39	TCGTCGGCAGCGTCAGATGTGTATAAGAGACAG TGAACAGTCATACCACCAAGTAA	GTCTCGTGGGCTCGGAGATGTGTATAAGAGACAG CAGCCAGTTTGATGACAGCA
OT40	TCGTCGGCAGCGTCAGATGTGTATAAGAGACAG TGCTTCTCAATATTCCCCAGC	GTCTCGTGGGCTCGGAGATGTGTATAAGAGACAG GGCCTCCAAAGTTCAGGGA
OT41	TCGTCGGCAGCGTCAGATGTGTATAAGAGACAG CCAACCACACTTCCCTCTCT	GTCTCGTGGGCTCGGAGATGTGTATAAGAGACAG CACCATAGGCACAAAGGTAGC
OT42	TCGTCGGCAGCGTCAGATGTGTATAAGAGACAG ACTGTACCATAACGATGCTAGC	GTCTCGTGGGCTCGGAGATGTGTATAAGAGACAG CCTTGATTCCGCCTTCTTTGT
OT43	TCGTCGGCAGCGTCAGATGTGTATAAGAGACAG GCCATGCCTAAAAGAAGCCA	GTCTCGTGGGCTCGGAGATGTGTATAAGAGACAG AGTTGACTTCTGTGCCCTTG
OT44	TCGTCGGCAGCGTCAGATGTGTATAAGAGACAG ATAGCAAGTGTGAAGGCCCA	GTCTCGTGGGCTCGGAGATGTGTATAAGAGACAG TGCTTCCAAACATCCCTTCC
OT45	TCGTCGGCAGCGTCAGATGTGTATAAGAGACAG GAGCCCTCCCATTGCCTAA	GTCTCGTGGGCTCGGAGATGTGTATAAGAGACAG AAGCACAGTTCTAGGCATATAGT
OT46	TCGTCGGCAGCGTCAGATGTGTATAAGAGACAG TAGAGATAGGCTTGGCAGGG	GTCTCGTGGGCTCGGAGATGTGTATAAGAGACAG AACTCGAATGCTCAACTGGC
OT47	TCGTCGGCAGCGTCAGATGTGTATAAGAGACAG AGGAAGATCACTGTCACCGT	GTCTCGTGGGCTCGGAGATGTGTATAAGAGACAG TCCCCTGTAATCCACTCACT
OT48	TCGTCGGCAGCGTCAGATGTGTATAAGAGACAG TGTCCTCCACTTCATTTCTTCAG	GTCTCGTGGGCTCGGAGATGTGTATAAGAGACAG ACCCTCTTCATTTCCCCTGG
OT49	TCGTCGGCAGCGTCAGATGTGTATAAGAGACAG GCAAGGAAGTGTTGTTCAGC	GTCTCGTGGGCTCGGAGATGTGTATAAGAGACAG TCTGACTCGTCTTTCCACAGA
OT50	TCGTCGGCAGCGTCAGATGTGTATAAGAGACAG GACTCTGGACTTGATGCCCT	GTCTCGTGGGCTCGGAGATGTGTATAAGAGACAG CCCACAGGCTCAACACTACA
OT51	TCGTCGGCAGCGTCAGATGTGTATAAGAGACAG CCACACCATGATCTGATTGCA	GTCTCGTGGGCTCGGAGATGTGTATAAGAGACAG GTCATCATCTCTGTTTCCGCT
OT52	TCGTCGGCAGCGTCAGATGTGTATAAGAGACAG AGGGTTAAGTGAGCAAGCAATG	GTCTCGTGGGCTCGGAGATGTGTATAAGAGACAG CCATGACTGCAATTCCACTGA
OT53	TCGTCGGCAGCGTCAGATGTGTATAAGAGACAG ACTCTACAGGGAAAACAGAACCA	GTCTCGTGGGCTCGGAGATGTGTATAAGAGACAG AAAGGTGCCTGTCAGTGGG
OT54	TCGTCGGCAGCGTCAGATGTGTATAAGAGACAG TGACAGAAAGAGGAGGAGACA	GTCTCGTGGGCTCGGAGATGTGTATAAGAGACAG CTCTGAAGGCTCTTGGGGAT
OT55	TCGTCGGCAGCGTCAGATGTGTATAAGAGACAG GGCACTTCTACATACAATGACT	GTCTCGTGGGCTCGGAGATGTGTATAAGAGACAG TCATTTTGGAGTCAGATTTAGGT
OT56	TCGTCGGCAGCGTCAGATGTGTATAAGAGACAG GGCATCACTCAGCTCTAAGG	GTCTCGTGGGCTCGGAGATGTGTATAAGAGACAG ACCAATCCCTCTGCACACA
OT57	TCGTCGGCAGCGTCAGATGTGTATAAGAGACAG GAGCGGGTCTTGAGCACC	GTCTCGTGGGCTCGGAGATGTGTATAAGAGACAG AGTATGAAATTCACATGGCCAC

MATERIALS AND METHODS

Vectors

The following adenine base editor-expressing plasmids were obtained from Addgene as a gift from Dr. Benjamin Kleinstiver:[1] SpG-ABE7.10 (pCMV-T7-ABEmax(7.10)-SpG-P2A-EGFP; Addgene plasmid # 140002; http://n2t.net/addgene:140002; RRID:Addgene_140002), SpG-ABE8.20 (pCMV-T7-ABE8.20m-nSpG-P2A-EGFP; Addgene plasmid # 185916; http://n2t.net/addgene:185916; RRID:Addgene_185916), SpG-ABE8e (pCMV-T7-ABE8e-nSpG-P2A-EGFP; Addgene plasmid # 185911; http://n2t.net/addgene:185911; RRID:Addgene_185911), and SpRY-ABE8e (pCMV-T7-ABE8e-nSpRY-P2A-EGFP; Addgene plasmid # 185912; http://n2t.net/addgene:185912; RRID:Addgene_185912). The following adenine base editor-expressing plasmid was obtained from Addgene as a gift from Dr. Nicole Gaudelli:[2] ABE8.8 (ABE8.8-m; Addgene plasmid # 136294; http://n2t.net/addgene:136294; RRID:Addgene 136294). The SpRY-ABE8.8 plasmid was made by combining elements of SpRY-ABE8e and ABE8.8 by standard molecular biology techniques. The following plasmid was obtained from Addgene as a gift from Dr. Joseph Jacobson:[3] iSpyMac (iSpyMac; Addgene plasmid # 155014; http://n2t.net/addgene:155014; RRID:Addgene 155014). The iSpyMac-ABE8.8 plasmid was made by combining elements of iSpyMac and ABE8.8 by standard molecular biology techniques. gRNAs were expressed from the pGuide plasmid (pGuide; Addgene plasmid # 64711; http://n2t.net/addgene:64711; RRID:Addgene_64711), with each of the following spacer sequences (plus an additional 5' G to facilitate U6 expression) cloned into the plasmid: PAH1, 5'-AAGGGCCAAGGTATTGTGGC-3'; PAH2, 5'-AGGGCCAAGGTATTGTGGCA-3'; PAH3, 5'-GGGCCAAGGTATTGTGGCAG-3'; PAH4, 5'-GGCCAAGGTATTGTGGCAGC-3'; PAH5, 5'-GCCAAGGTATTGTGGCAGCA-3'; and PAH6, 5'-CCAAGGTATTGTGGCAGCAA-3'. pmaxGFP was purchased from Lonza. Any of the newly generated plasmids is readily available from the authors via a Transfer of Research Material agreement with the University of Pennsylvania.

RNA Production

Guide RNAs were chemically synthesized under solid phase synthesis conditions by a commercial supplier (Agilent) with end-modifications as well as heavy 2'-O-methylribosugar modification as previously described.[4] 100-mer PAH4 gRNA: 5'-mG*mG*mC*CAAGGUAUUGUGGCAGCGUUUUAGAmGmCmUmAmGmAmAmAmUmAmGmCAAGUUAAAAUAAGGCUAGUCCGUUAUCAmAmCmUmUmGmAmAmAmAmAmGmUmGmGmCmAmCmCmGmAmGmUmCmGmGmUmGmCmU*mU*mU*mU-3', where "m" and * respectively indicate 2'-O-methylation and phosphorothioate linkage. 120-mer P56 pegRNA: 5'-mA*mC*mU*UUGCUGCCACAAUACCUGUUUUAGAmGmCmUmAmGmAmAmAmUmAmGmCAAGUUAAAAUAAGGCUAGUCCGUUAUCAmAmCmUmUmGmAmAmAmAmAmGmUmGmGmCmAmCmCmGmAmGmUmCmGmGmUmGmCGAGAAGGGACGUGGUAUUGUG*mG*mC*mA-3'. 100-mer N19 ngRNA: 5'-mU*mG*mA*GAAGGGACGUGGUAUUGGUUUUAGAmGmCmUmAmGmAmAmAmUmAmGmCAAGUUAAAAUAAGGCUAGUCCGUUAUCAmAmCmUmUmGmAmAmAmAmAmGmUmGmGmCmAmCmCmGmAmGmUmCmGmGmUmGmCmU*mU*mU*mU-3'. SpRY-ABE8.8, PEmax,[5] and GFP mRNAs were produced via in vitro transcription (IVT) and purification. In brief, a plasmid DNA template containing a codon-optimized gene coding sequence and a 3' polyadenylate sequence was linearized. An IVT reaction containing linearized DNA template, T7 RNA polymerase, NTPs, and cap analog was performed to produce mRNA containing N1-methylpseudouridine. After digestion of the DNA template with DNase I, the mRNA product underwent purification and buffer exchange, and the purity of the final mRNA product was assessed with spectrophotometry and capillary gel electrophoresis. Elimination of double-stranded RNA contaminants was assessed using dot blots and transfection into human dendritic cells.

Endotoxin content was measured using a chromogenic Limulus amebocyte lysate (LAL) assay; all assays were negative.

LNP Formulation

LNPs were formulated as previously described,[4,6] with the lipid components (SM-102, 1,2-distearoyl-sn-glycero-3-phosphocholine, cholesterol, and PEG-2000 at molar ratios of 50:10:38.5:1.5) being rapidly mixed with an aqueous buffer solution containing (1) SpRY-ABE8.8 mRNA and PAH4 gRNA in a 1:1 ratio by weight, (2) PEmax mRNA, P56 pegRNA, and N19 ngRNA in a 3:2:1 ratio by weight, or (3) GFP mRNA in 25 mM sodium acetate (pH 4.0), with an N:P ratio of 5.6. The resulting LNP formulation was subsequently dialyzed against sucrose-containing buffer, concentrated using Amicon Ultra-15 mL Centrifugal Filter Units (Millipore Sigma), sterile-filtered using 0.2-μm filters, and frozen until use.

Culture and Transfection of HuH-7 Cells

The methods were similar to those previously described.[4] *PAH* c.1222C>T homozygous HuH-7 cell lines were generated as described elsewhere.[7] HuH-7 cells were maintained in Dulbecco's modified Eagle's medium (containing 4 mM L-glutamine and 1 g/L glucose) with 10% fetal bovine serum and 1% penicillin/streptomycin at 37°C with 5% CO_2. HuH-7 cells were seeded on 6-well plates (Corning) at 3.5×10^5 cells per well. At 16–24 hours after seeding, cells were transfected at approximately 80–90% confluency with 9 μL TransIT®-LT1 Transfection Reagent (MIR2300, Mirus), 2 μg base editor plasmid, and 1 μg gRNA plasmid per well according to the manufacturer's instructions; alternatively, GFP-expressing pmaxGFP plasmid was transfected. LNPs were added at various doses (quantified by the total amount of RNA within the LNPs) directly to the media. Cells for base editing experiments were cultured for 72 hours after transfection, and then media were removed, cells were washed with 1× DPBS (Corning), and genomic DNA was isolated using the DNeasy Blood and Tissue Kit (QIAGEN) according to the manufacturer's instructions. GFP-expressing cells were cultured for 48 hours after transfection, and then the cells were trypsinized and resuspended into 200 μL 1× DPBS with 0.1% BSA; flow cytometry for GFP positivity was performed on a CytoFLEX S Flow Cytometer (Beckman Coulter) and quantified with FlowJo v10.9.

Generation of Humanized PKU Mouse Model

The methods were similar to those previously described.[4] The PKU mouse model with humanized *Pah* c.1222C>T alleles was generated using in vitro transcribed Cas9 mRNA, a synthetic gRNA (spacer sequence 5'-AGCGAACGGAGAAGGGCCGG-3') (Synthego), and a synthetic single-strand DNA oligonucleotide (Integrated DNA Technologies) with homology arms matching the target site and harboring the c.1222C>T variant and synonymous variants (bold with underline): 5'-AAAAGCCACTT
GGAACTCCTCCAGGATAACCTGTCTTTAAATGGTGTCCTTCACTGGGGTCCTTGGTTTTGGT
TTCAGGA**AC**TTTGCTGCCACAAT**A**CC**TT**GGCCCTTCTC**A**GTTCGCTA**C**GACCCCTACACTCA
AAGGGTTGAGGTCCTGGACAATACTCAGCAGTTGAAGATTTTAGCTGACTCCATTAATAGT
AAGT-3'. The mixture of the 3 components was injected into cytoplasm of fertilized oocytes from C57BL/6J mice at the Penn Vet Transgenic Mouse Core (https://www.vet.upenn.edu/research/core-resources-facilities/transgenic-mouse-core). Genomic DNA samples from founders were screened for knock-in of the desired sequence in the *Pah* locus via homology-directed repair. Founders with the humanized c.1222C>T allele were bred through two generations to obtain homozygous mice. This humanized PKU mouse model is readily available from the authors via a Transfer of Research Material agreement with the University of Pennsylvania.

Mouse Studies

The methods were similar to those previously described.[4] All procedures used in mouse studies were approved by the Institutional Animal Care and Use Committee at the University of Pennsylvania (protocol #805887), where the studies were performed, and were consistent with local, state, and federal regulations as applicable, including the National Institutes of Health Guide for the Care and Use of Laboratory Animals. Mice were maintained on a 12-hour light/12-hour dark cycle, with a temperature range of 65°F to 75°F and a humidity range of 40% to 60%, and were fed ad libitum with a chow diet (LabDiet, Laboratory Autoclavable Rodent Diet 5010). Homozygous humanized PKU mice, as well as heterozygous humanized non-PKU mice, were generated as littermates/colonymates via timed breeding, in some cases using wild-type C57BL/6J mice (stock no. 000664; RRID:IMSR_JAX:000664) obtained from The Jackson Laboratory. Genotyping was performed using PCR amplification from genomic DNA samples (prepared from clipped tails/ears) followed by Sanger sequencing or by next-generation sequencing. Age-matched female and male colonymates were used for experiments at approximately 8 weeks of age with random assignment of animals to various experimental groups when applicable, and with collection and analysis of data performed in a blinded fashion when possible. LNPs were administered to the mice at either 5 mg/kg or 2.5 mg/kg doses, diluted with Dulbecco's phosphate-buffered saline, via retro-orbital injection under anesthesia with 1%-2% inhaled isoflurane; similar volumes of Dulbecco's phosphate-buffered saline were administered to vehicle control mice. In short-term studies, mice were euthanized at 1 week after treatment, and 8 liver samples (2 from each lobe) were obtained on necropsy and processed with the DNeasy Blood and Tissue Kit (QIAGEN) as per the manufacturer's instructions to isolate genomic DNA. Euthanasia in all instances was achieved via terminal inhalation of carbon dioxide followed by secondary euthanasia through cervical dislocation or decapitation, consistent with the 2020 American Veterinary Medical Association Guidelines on Euthanasia. Next-generation sequencing results from the liver samples were averaged to provide quantification of whole-liver editing. Blood samples were collected via the tail tip at various timepoints (pre-treatment, day 1, day 2, day 3, day 4 or day 7), with daily measurements in the early afternoon to account for diurnal variation in blood phenylalanine levels, or via retro-orbital bleeding with capillary tubes at various timepoints (pre-treatment, 4 hours, and 24 hours) to assess cytokine and chemokine levels.

Measurement of Blood Analytes

Some methods were similar to those previously described.[4] The blood phenylalanine levels were measured by an enzymatic method using the Phenylalanine Assay Kit (MAK005, Millipore Sigma) according to the manufacturer's instructions. Plasma samples were deproteinized with a 10 kDa MWCO spin filter (CLS431478-25EA, Millipore Sigma) and pre-treated with 5 µL of tyrosinase for 10 minutes at room temperature prior to start of the assay. Reaction mixes were made according to the manufacturer's instructions, and the fluorescence intensity of each sample was measured (λ_{ex} = 535/λ_{em} = 587 nm). Aspartate aminotransferase (AST) (ab263882, Abcam) and alanine aminotransferase (ALT) (MAK052-1KT, Millipore Sigma) activity were measured according to the manufacturers' instructions. AST measurements, determined in units of pg/mL, were converted to units of IU/L via normalization to reference samples. The expression profile of cytokines and chemokines in mouse serum samples was determined using the BioLegend anti-virus response panel [13-plex bead-based assay for the quantification of interferons (α, β, γ), interleukins (1β, 6, 10, 12p70), and chemokines (MCP-1, RANTES, CXCL-1, IP-10, TNF-α, and GM-CSF)] according to the manufacturer's instructions. Briefly, kit components were thawed and reconstituted as needed. Standards were prepared by 1:4 serial dilution in assay buffer. Serum samples were diluted 1:3 in Matrix A. Filter bottom plates were prewetted with wash buffer before addition of standards and samples. 25 µL of each standard and sample was added to

the plates in duplicates and mixed with 25 µL of premixed beads solution. Plates were incubated with shaking at 500 rpm for 2 hours and washed twice, followed by addition of 25 µL detection antibodies and incubation at 500 rpm for 1 hour. Without washing, 25 µL SA-PE was added to each well and incubated with shaking at 500 rpm for 30 minutes. Plates were washed twice, and beads were resuspended in 150 µL wash buffer, transferred to FACS tubes, and acquired on an BD LSR flow cytometer. The standard curve was used to determine the concentrations of each analyte in the serum samples using a 5PL fitting.

In Silico Nomination of Candidate Off-target Sites

Sites with up to 2 mismatches, or up to 1 mismatch plus up to 2 DNA or RNA bulges, compared to the *PAH* on-target site (i.e., compared to the PAH4 protospacer sequence without any regard for PAM sequence) were identified with Cas-Designer v1.2.[8] The results of next-generation sequencing (see below) for these sites are listed in Table S2.

Next-generation Sequencing

The methods were similar to those previously described.[4] PCR reactions were performed using NEBNext Polymerase (NEB) using the primer sets listed in Table S3, designed with Primer3 v4.1.0 (https://primer3.ut.ee/). The following program was used for all genomic DNA PCRs: 98°C for 20 seconds, 35× (98°C for 20 seconds, 57°C for 30 seconds, 72°C for 10 seconds), 72°C for 2 minutes. PCR products were visualized via capillary electrophoresis (QIAxcel, QIAGEN) and then purified and normalized via an NGS Normalization 96-Well Kit (Norgen Biotek Corporation). A secondary barcoding PCR was conducted to add Illumina barcodes (Nextera XT Index Kit V2 Set A and/or Nextera XT Index Kit V2 Set D) using ≈15 ng of first-round PCR product as template, followed by purification and normalization. Final pooled libraries were quantified using a Qubit 3.0 Fluorometer (Thermo Fisher Scientific) and then after denaturation, dilution to 10 pM, and supplementation with 15% PhiX, underwent single-end or paired-end sequencing on an Illumina MiSeq System. The amplicon sequencing data were analyzed with CRISPResso2 v2[9] and scripts to quantify editing. For on-target editing, A-to-G editing was quantified at the site of the c.1222C>T variant (position 5 of the PAH4 protospacer sequence) and at the site of the potential bystander adenines (positions –2, –1, 6, and 10 of the PAH4 protospacer sequence). For candidate off-target sites, A-to-G editing was quantified throughout the editing window (positions 1 to 10 of the protospacer sequence).

Data Analysis

GFP positivity and sequencing data were analyzed as described above. Other data were collected and analyzed using GraphPad Prism v10.0.2.

References

1. Walton, R.T., Christie, K.A., Whittaker, M.N., and Kleinstiver, B.P. (2020). Unconstrained genome targeting with near-PAMless engineered CRISPR-Cas9 variants. Science *368*, 290–296. https://doi.org/10.1126/science.aba8853.

2. Gaudelli, N.M., Lam, D.K., Rees, H.A., Solá-Esteves, N.M., Barrera, L.A., Born, D.A., Edwards, A., Gehrke, J.M., Lee, S.J., Liquori, A.J., et al. (2020). Directed evolution of adenine base editors with increased activity and therapeutic application. Nat. Biotechnol. *38*, 892–900. https://doi.org/10.1038/s41587-020-0491-6.

3. Chatterjee, P., Lee, J., Nip, L., Koseki, S.R.T., Tysinger, E., Sontheimer, E.J., Jacobson, J.M., and Jakimo, N. (2020). A Cas9 with PAM recognition for adenine dinucleotides. Nat. Commun. *11*, 2474. https://doi.org/10.1038/s41467-020-16117-8.

4. Brooks, D.L., Carrasco, M.J., Qu, P., Peranteau, W.H., Ahrens-Nicklas, R.C., Musunuru, K., Alameh, M.-G., and Wang, X. (2023). Rapid and definitive treatment of phenylketonuria in variant-humanized mice with corrective editing. Nat. Commun. *14*, 3451. https://doi.org/10.1038/s41467-023-39246-2.

5. Chen, P.J., Hussmann, J.A., Yan, J., Knipping, F., Ravisankar, P., Chen, P.F., Chen, C., Nelson, J. W., Newby, G.A., Sahin, M., et al. (2021). Enhanced prime editing systems by manipulating cellular determinants of editing outcomes. Cell *184*, 5635–5652.e29. https://doi.org/10.1016/j.cell.2021.09.018.

6. Carrasco, M.J., Alishetty, S., Alameh, M.G., Said, H., Wright, L., Paige, M., Soliman, O., Weissman, D., Cleveland, T.E., 4th, Grishaev, A., et al. (2021). Ionization and structural properties of mRNA lipid nanoparticles influence expression in intramuscular and intravascular administration. Commun. Biol. *4*, 956. https://doi.org/10.1038/s42003-021-02441-2.

7. Brooks, D.L., Whittaker, M.N., Qu, P., Musunuru, K., Ahrens-Nicklas, R.C., and Wang, X. (2023). Efficient in vivo prime editing corrects the most frequent phenylketonuria variant, associated with high unmet medical need. Am. J. Hum. Genet. https://doi.org/10.1016/j.ajhg.2023.10.005.

8. Bae, S., Park, J., and Kim, J.S. (2014). Cas-OFFinder: a fast and versatile algorithm that searches for potential off-target sites of Cas9 RNA-guided endonucleases. Bioinformatics *30*, 1473–1475. https://doi.org/10.1093/bioinformatics/btu048.

9. Clement, K., Rees, H., Canver, M.C., Gehrke, J.M., Farouni, R., Hsu, J.Y., Cole, M.A., Liu, D.R., Joung, J.K., Bauer, D.E., et al. (2019). CRISPResso2 provides accurate and rapid genome editing sequence analysis. Nat. Biotechnol. *37*, 224–226. https://doi.org/10.1038/s41587-019-0032-3.
