# Supplementary material for: A base editing strategy using mRNA-LNPs for in vivo correction of the most frequent phenylketonuria variant
Source: HGG Adv. 2023 Nov 2;5(1):100253. doi: 10.1016/j.xhgg.2023.100253 (PMC10800763; doi:10.1016/j.xhgg.2023.100253)
Supplement: Document S2. Article plus supplemental information [file mmc2.pdf]

HGG
Advances

REPORT

A base editing strategy using mRNA-LNPs for *in vivo* correction of the most frequent phenylketonuria variant

Dominique L. Brooks,[1,2,3] Madelynn N. Whittaker,[1,2,3,4] Hooda Said,[5] Garima Dwivedi,[6] Ping Qu,[1,2,3] Kiran Musunuru,[1,2,3,11,*] Rebecca C. Ahrens-Nicklas,[3,7,8,11] Mohamad-Gabriel Alameh,[5,9,10,11] and Xiao Wang[1,2,3,11]

Summary

The c.1222C>T (p.Arg408Trp) phenylalanine hydroxylase (*PAH*) variant is the most frequent cause of phenylketonuria (PKU), an autosomal recessive disorder characterized by accumulation of blood phenylalanine (Phe) to neurotoxic levels. Here we devised a therapeutic base editing strategy to correct the variant, using prime-edited hepatocyte cell lines engineered with the c.1222C>T variant to screen a variety of adenine base editors and guide RNAs *in vitro*, followed by assessment in c.1222C>T humanized mice *in vivo*. We found that upon delivery of a selected adenine base editor mRNA/guide RNA combination into mice via lipid nanoparticles (LNPs), there was sufficient *PAH* editing in the liver to fully normalize blood Phe levels within 48 h. This work establishes the viability of a base editing strategy to correct the most common pathogenic variant found in individuals with the most common inborn error of metabolism, albeit with potential limitations compared with other genome editing approaches.

Phenylketonuria (PKU [MIM: 261600]) is a disorder of phenylalanine (Phe) metabolism wherein deficiency of phenylalanine hydroxylase (PAH) results in elevated blood Phe levels. Optimal management of PKU requires strict, lifelong monitoring and control of blood Phe levels to maintain them within the recommended range of 120–360 μmol/L.[1] When not adequately treated, blood Phe levels can exceed 1200 μmol/L, and as a result, PKU individuals can develop irreversible neurological impairment and neuropsychiatric issues. Although there are several treatment options to regulate blood Phe levels within the recommended range—including a low-Phe diet, an oral medication that serves as a cofactor of PAH (sapropterin), and a daily injectable enzyme substitution therapy (pegvaliase)—more than 70% of adults with PKU are noncompliant with treatment guidelines due to challenges associated with adherence and therapy responsiveness.[2]

The most frequently occurring pathogenic *PAH* variant worldwide is the c.1222C>T (p.Arg408Trp) variant (RefSeq: NM_000277.3), particularly prevalent in European countries and the United States.[3] We have found in a parallel study[4] being simultaneously published in *The American Journal of Human Genetics* that most individuals with *PAH* c.1222C>T variants experience chronic, severe Phe elevations, reflecting in part the limitations of the existing treatment options.[5,6] Genome editing offers the potential of a one-time curative therapy to permanently normalize blood Phe levels. Base editing is particularly attractive because it can precisely and efficiently correct pathogenic variants.[7,8] In a recent study, we found that base editing could rapidly (within 48 h) and definitively treat a humanized mouse model of PKU with the *PAH* c.842C>T (p.Pro281Leu) variant when intravenously delivered in the form of mRNA and guide RNA (gRNA) encapsulated in lipid nanoparticles (LNPs) targeting the liver, where *PAH* mRNA is specifically expressed.[9] As little as 10% correction of the gene is sufficient to normalize blood Phe levels.

In principle, because the *PAH* c.1222C>T variant results from a C→T change on the sense strand, the variant is amenable to correction by an adenine base editor introducing an A→G change at the same position on the antisense strand. However, upon inspection of the genomic site of the variant (Figure 1A), two substantial impediments to therapeutic base editing become evident. First, the position of the target adenine does not lie within the editing window (roughly from positions 3 to 9 of the protospacer sequence) for any protospacer that has an NGG

[1]Cardiovascular Institute, Perelman School of Medicine at the University of Pennsylvania, Philadelphia, PA 19104, USA; [2]Division of Cardiovascular Medicine, Department of Medicine, Perelman School of Medicine at the University of Pennsylvania, Philadelphia, PA 19104, USA; [3]Department of Genetics, Perelman School of Medicine at the University of Pennsylvania, Philadelphia, PA 19104, USA; [4]Department of Bioengineering, University of Pennsylvania, Philadelphia, PA 19104, USA; [5]Department of Bioengineering, George Mason University, Fairfax, VA 22030, USA; [6]Division of Infectious Diseases, Department of Medicine, Perelman School of Medicine at the University of Pennsylvania, Philadelphia, PA 19104, USA; [7]Metabolic Disease Program, Division of Human Genetics, Department of Pediatrics, Children's Hospital of Philadelphia, Philadelphia, PA 19104, USA; [8]Department of Pediatrics, Perelman School of Medicine at the University of Pennsylvania, Philadelphia, PA 19104, USA; [9]Department of Pathology and Laboratory Medicine, Children's Hospital of Philadelphia, Philadelphia, PA 19104, USA; [10]Department of Pathology and Laboratory Medicine, Perelman School of Medicine at the University of Pennsylvania, Philadelphia, PA 19104, USA
[11]Senior authors
*Correspondence: kiranmusunuru@gmail.com
https://doi.org/10.1016/j.xhgg.2023.100253.
© 2023 The Author(s). This is an open access article under the CC BY license (http://creativecommons.org/licenses/by/4.0/).

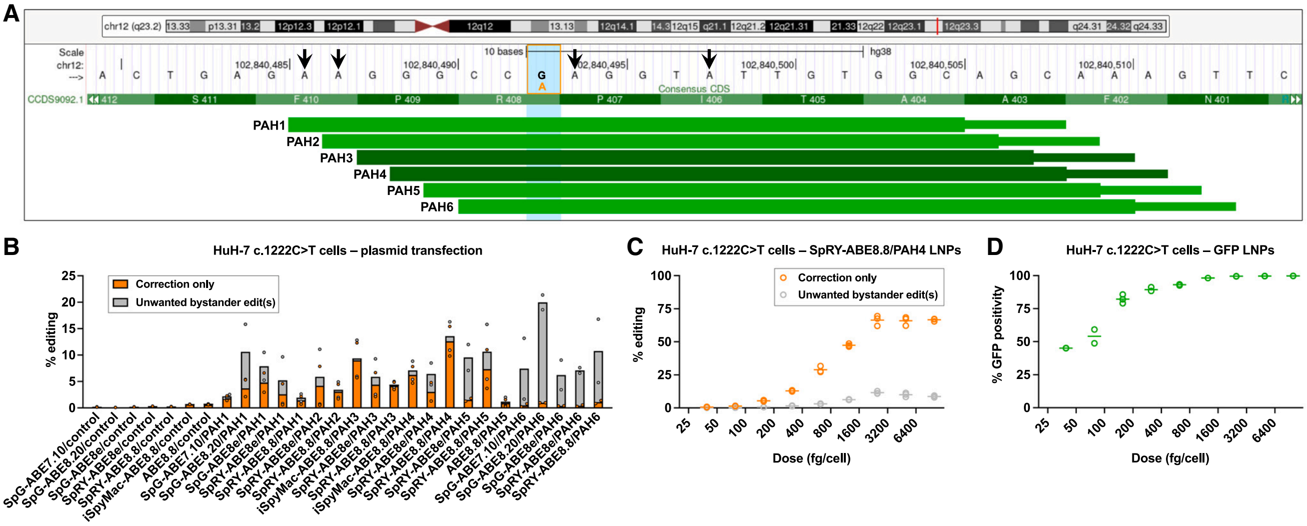

Figure 1. Base editing to correct *PAH* c.1222C>T variant in human hepatocytes *in vitro*
(A) Schematic of the genomic site of the *PAH* c.1222C>T variant, adapted from the UCSC Genome Browser (GRCh38/hg38). The vertical blue bar outlined by the orange box indicates the G altered to A (in orange) by the variant on the antisense strand. The arrows indicate the sites of potential bystander editing. The horizontal green bars indicate protospacer (thick) and PAM (thin) sequences targeted by the PAH1 through PAH6 gRNAs.
(B) Corrective *PAH* c.1222C>T editing (determined from genomic DNA) following transfection of cells with plasmids encoding adenine base editor/gRNA combinations (n = 2 biological replicates, one each from two *PAH* c.1222C>T homozygous HuH-7 cell lines; controls, n = 1), calculated as the proportion of aligned sequencing reads with the indicated type of edits. "Correction only" refers to reads in which the c.1222C>T adenine variant is edited to guanine, with or without base editing of the adjacent synonymous adenine, with no base editing of any other adenines; "unwanted bystander editing" refers to reads in which the c.1222C>T adenine variant is edited to guanine, along with base editing of one or more nonsynonymous adenines.
(C) Dose-response study with *PAH* c.1222C>T homozygous HuH-7 cells treated with SpRY-ABE8.8/PAH4 LNPs (n = 3 biological replicates).
(D) Dose-response study with *PAH* c.1222C>T homozygous HuH-7 cells treated with GFP LNPs (n = 2 to 3 biological replicates).

protospacer-adjacent motif (PAM), the preferred PAM for standard *Streptococcus pyogenes* Cas9 (SpCas9)-containing editors. Accordingly, one would need to use either SpCas9 variants with altered PAM preferences or non-Sp Cas9 proteins with non-NGG PAM preferences, likely with reduced editing efficiency even if there is optimal spacing of the target adenine within the editing window. Second, there are four nearby non-target adenines that could potentially be edited in conjunction with the target adenine, resulting in bystander editing. The adenine in the adjacent position downstream of the target adenine, if converted to guanine, would represent a synonymous edit (codon CCT to codon CCC, both encoding proline) unlikely to have a functional consequence. In contrast, the adenine located five positions downstream of the target adenine, if converted to guanine, would represent a nonsynonymous edit (codon ATA to codon ACA) resulting in the *PAH* c.1217T>C (p.Ile406Thr) variant, which has been reported to occur in PKU individuals and thus is likely to compromise PAH function.[10,11] Similarly, the adenines located six and seven positions upstream of the target adenine, if singly or both converted to guanine, would represent nonsynonymous edits (codon TTC to codon CTC, TCC, or CCC) that would change the phenylalanine in amino acid position 410 to leucine, serine, or proline. Thus, achieving the desired on-target editing without undesired bystander editing in either direction could prove challenging.

In our parallel study,[4] we had already used prime editing to generate clonal HuH-7 human hepatoma cell lines (as a proxy for human hepatocytes) homozygous for the *PAH* c.1222C>T variant. Using two of the clonal lines, we evaluated an array of adenine base editors and gRNAs for editing activity at the site of the variant, assessing for both on-target editing and unwanted bystander editing. We used four different Cas9 variants: standard SpCas9 (which prefers NGG PAMs but can also engage NAG and NGA PAMs, albeit with less activity), iSpyMac (which prefers NAA PAMs),[12] SpG (which generally engages NGN PAMs),[13] and SpRY (which being near-PAMless can engage a broad range of sequences).[13] We used four different adenosine deaminase domains: ABE7.10 (seventh-generation, less activity),[8] ABE8e (eighth-generation, more activity, broadest editing window),[14] ABE8.20 (eighth-generation, more activity, intermediate editing window),[15] and ABE8.8 (eighth-generation, more activity, narrowest editing window).[15] We used six different gRNAs (termed PAH1 through PAH6) tiling the site of the variant, with distinct PAMs (AGC, GCA, CAA, AAA, AAG, and AGT, respectively) (Figure 1A). Via plasmid transfection, we tested a subset of combinations of Cas9 variant, adenosine deaminase domain, and gRNA, tailored to the position of the variant within the editing window and to the available PAM (Figures 1B and S1). To serve as a reference for transfection efficiency, we transfected a plasmid encoding green fluorescent protein (GFP) and observed

mean 85% GFP positivity as determined by flow cytometry. In c.1222C>T homozygous HuH-7 cells, SpRY-ABE8.8/PAH4 yielded the best combination of higher on-target editing and lower bystander editing, reflecting that PAH4 places the variant adenine in the middle of the window (protospacer position 5), ABE8.8 has a narrow window that limits bystander editing on either side of the variant adenine, and SpRY provides more activity than iSpyMac with the same PAH4 gRNA with its AAA PAM (in contrast to the PAH3 gRNA, for which iSpyMac outperformed SpRY). We formulated LNPs with SpRY-ABE8.8 mRNA and synthetic PAH4 gRNA and performed a dose-response experiment in c.1222C>T homozygous HuH-7 cells, observing as high as mean 80% overall editing (with as high as mean 12% unwanted bystander editing) at the highest LNP doses and a half maximal effective concentration (EC_{50}) of 750 fg/cell (Figures 1C and S2). To serve as a reference for transfection efficiency, we also formulated LNPs with GFP mRNA and performed a similar dose-response experiment (Figure 1D).

To test the SpRY-ABE8.8/PAH4 combination *in vivo*, we used CRISPR-Cas9 targeting in mouse embryos to generate a humanized PKU model with the *PAH* c.1222C>T (p.Arg408Trp) variant (hereafter referred to as R408W mice) in the C57BL/6J background, in which we replaced a small portion of the endogenous mouse *Pah* exon 12 with the orthologous human sequence spanning the PAH4 gRNA protospacer/PAM sequences and containing the c.1222C>T variant (Figure S3A). Upon breeding the humanized c.1222C>T allele to homozygosity, we observed phenotypes consistent with PKU, including elevated blood Phe levels, mild hypopigmentation (resulting from reduced melanin synthesis due to decreased tyrosine levels because of deficient PAH activity), and reduced body weight (Figures S3B and S3C). (In our parallel study,[4] we generated a different homozygous R408W mouse model via homologous recombination in mouse embryonic stem cells, replacing the entirety of *Pah* exon 12 as well as flanking intronic regions with the orthologous human sequence, but we did not use that alternative model for this base editing study.)

We treated homozygous R408W PKU mice, approximately 8 weeks of age and with baseline blood Phe levels in the 1,000–1,500 μmol/L range, with SpRY-ABE8.8/PAH4 LNPs at two dose levels. PKU mice that received a single 5-mg/kg LNP dose experienced normalization of blood Phe levels by 48 h after treatment (mean 118 μmol/L, 90% reduction from baseline), and PKU mice that received a single 2.5-mg/kg LNP dose had substantial, though somewhat less, reduction of blood Phe levels by 48 h after treatment (mean 185 μmol/L, 86% reduction from baseline) (Figure 2A). All mice in both dose groups achieved blood Phe levels less than 125 μmol/L by 7 days after treatment. Vehicle-treated age-matched homozygous R408W PKU mice maintained elevated blood Phe levels during the same time course, and vehicle-treated age-matched heterozygous R408W non-PKU mice generally had blood Phe levels less than 125 μmol/L. There were no alanine aminotransferase (ALT) abnormalities in any of the mice over the same time period, with slight rises in aspartate aminotransferase (AST) levels at 1 day after treatment only in the mice that received the 5-mg/kg LNP dose, remaining within the normal range (Figures S4A and S4B). Out of a panel of 13 cytokines and chemokines, LNP treatment resulted in transient increases in C-X-C motif chemokine ligand 1, tumor necrosis factor-α, monocyte chemoattractant protein 1, interleukin (IL)-1β, interferon inducible protein 10, interferon-α, and IL-6 relative to vehicle treatment at 4 h after treatment, with resolution by 24 h after treatment (Table S1). Upon necropsy at 1 week after treatment, next-generation sequencing of genomic DNA from whole-liver samples to determine corrective editing activity showed mean 29% desired on-target editing and 4% undesired bystander editing in the higher-dosed mice and mean 26% desired on-target editing and 3% undesired bystander editing in the lower-dosed mice (Figures 2B and 2C). Liver histology showed no evidence of pathology (Figure S4C).

A potential liability of the use of a SpRY Cas9 variant is a higher burden of off-target editing due to its near-PAMless nature allowing it to engage a far broader range of genomic sites than standard Cas9. To evaluate off-target editing by SpRY-ABE8.8/PAH4, we generated a list of 57 candidate genomic sites nominated by *in silico* prediction based on sequence similarity to the on-target *PAH* site—including sites with up to one protospacer mismatch plus up to two DNA or RNA bulges, or with up to two protospacer mismatches with no bulges, with no constraint on the PAM sequence. Next-generation sequencing of targeted PCR amplicons from genomic DNA extracted from SpRY-ABE8.8/PAH4 LNP-treated c.1222C>T homozygous HuH-7 cells, versus control cells, revealed just one site with very low off-target base editing (net editing of 0.11%), within a very large intron of *ILRAPL2* and unlikely to be of biological significance (Figure 3).

In our parallel study,[4] we used an optimized prime editing configuration, delivered via dual adeno-associated viral (AAV) vectors, to correct the *PAH* c.1222C>T variant in humanized mice. A high dose of AAV treatment resulted in mean ≈40% corrective editing and no bystander editing, a better result than with SpRY-ABE8.8/PAH4 LNPs as reported in this study. To perform a head-to-head comparison between base editing and prime editing mediated by LNP delivery, we formulated LNPs with PEmax mRNA, synthetic prime editing guide RNA (pegRNA), and synthetic nicking guide RNA (ngRNA), analogous to the lead pegRNA/ngRNA combination used in the dual AAV configuration.[4] Upon treatment of homozygous R408W PKU mice with a 5-mg/kg dose of these LNPs, there were no significant changes in blood Phe levels, and there was minimal whole-liver editing (<1%) upon necropsy (Figure S5). Possible reasons for the lack of efficacy of the prime editing LNPs made in the same way as the base editing LNPs include the need to synthesize the substantially longer mRNA (PEmax, ≈6.7 kb) and pegRNA (120 nucleotides)

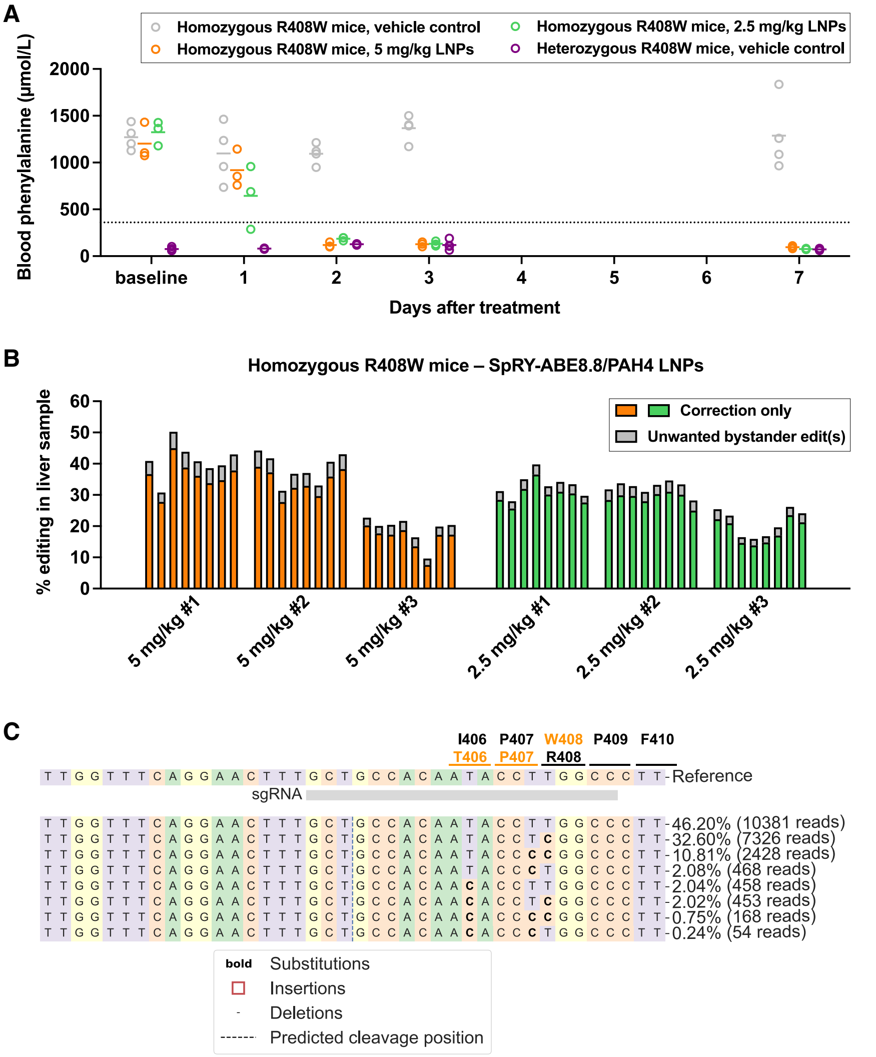

Figure 2. Base editing to correct *PAH* c.1222C>T variant in humanized mice
(A) Changes in blood phenylalanine levels in homozygous PKU mice following treatment with 5-mg/kg dose of SpRY-ABE8.8/PAH4 LNPs (n = 3 animals) or with 2.5-mg/kg dose of LNPs (n = 3 animals), comparing levels at various timepoints up to 7 days following treatment to levels in vehicle-treated homozygous PKU control (n = 4 animals) and vehicle-treated heterozygous non-PKU control (n = 4 animals) age-matched (approximately 8 weeks of age) colonymates (one blood sample per time point).
(B) Corrective *PAH* c.1222C>T editing (determined from genomic DNA) in each of eight liver samples (two samples each from the four lobes) from each treated mouse, calculated as the proportion of aligned sequencing reads with the indicated type of edits. “Correction only” refers to reads in which the c.1222C>T adenine variant is edited to guanine, with or without base editing of the adjacent synonymous adenine, with no base editing of any other adenines; “unwanted bystander editing” refers to reads in which the c.1222C>T adenine variant is edited to guanine, along with base editing of one or more nonsynonymous adenines.
(C) Standard CRISPResso output for a liver sample from the LNP-treated homozygous PKU mouse with the highest level of editing. The codons in the vicinity of the c.1222C>T variant site are indicated; the top-listed amino acid is the baseline identity of the codon, and the bottom-listed amino acid is the one that results from base editing of the adenine in the codon. Lines in graphs = mean values.

compared with the mRNA (SpRY-ABE8.8, ≈5.0 kb) and standard gRNA (100 nucleotides) used for base editing, which presents challenges of scale and purity; the need to encapsulate the larger mRNA along with two guide RNAs rather than one guide RNA within LNPs; slower kinetics of prime editing compared with base editing, which might require more prolonged expression of the editor in cells than that provided by the use of standard mRNA-LNPs; and reduced affinity of the pegRNA for the prime editing protein, due to auto-inhibition of the pegRNA.[16] While prime editing has more flexibility of site selection and avoids the bystander editing observed with base editing, its dependence on high doses of AAV vectors for effective *in vivo* delivery currently makes it a less favorable therapeutic option than LNP-mediated base editing, an approach that is already being evaluated in clinical trials.[17]

We acknowledge the limitations of this study as well as the base editing approach reported here. We did not evaluate all possible combinations of the existing catalogs of PAM-altered Cas9 variants, adenosine deaminase domains, and gRNAs, and there likely are combinations that would have equal or better corrective editing efficiency while also having less bystander editing and more favorable off-target profiles, making them more credible therapeutic candidates. Alternative Cas9 variants include engineered SpCas9 variants preferring NGA PAMs,[18] NGCG PAMs,[18] NGK PAMs,[19] NGN PAMs,[20] or NRNH PAMs,[21] as well as a wide variety of naturally occurring and engineered Cas9 proteins originating from species other than *Streptococcus pyogenes*. Recognizing that SpRY-ABE8.8/PAH4 is unlikely to be the optimal adenine base editor for correction of the *PAH* c.1222C>T variant, we did not perform an exhaustive analysis of its off-target editing. We also did not evaluate for the possibility of gRNA-independent off-target editing by SpRY-ABE8.8, wherein the deaminase domain of the base editor can spuriously deaminate genomic nucleic acid sequences independently of the Cas9 protein; notably, prior studies have suggested that this phenomenon is minimized when eighth-generation adenine base editors are delivered as mRNAs.[15,22] When SpRY-ABE8.8/PAH4 base editing for correction of the *PAH* c.1222C>T variant is compared against a similar base editing approach for correction of the *PAH* c.842C>T (p.Pro281Leu) variant,[9] also a frequent (albeit much less frequent) PKU variant, the former had

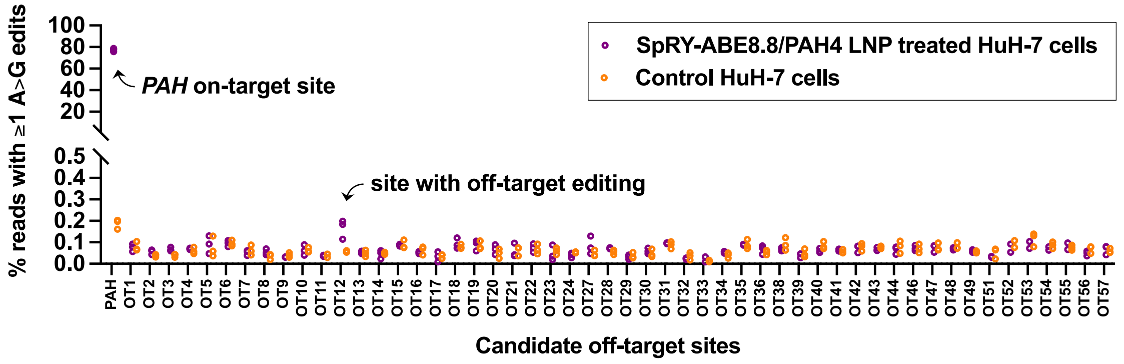

Figure 3. Assessment of off-target editing
On-target or off-target editing at top *in silico*-nominated candidate sites calculated as the proportion of aligned sequencing reads with ≥1 adenine base edited to guanine within the editing window at each site in *PAH* c.1222C>T homozygous HuH-7 cells that underwent treatment with SpRY-ABE8.8/PAH4 LNPs at a dose of 10,000 fg/cell (n = 3 treated and 3 untreated biological replicates), the highest dose shown in Figure 1C. Sites with unsuccessful sequencing are omitted. Refer to Table S2 for candidate site sequences and numerical values.

substantially less potency in HuH-7 cells (EC_{50} of 750 fmol/cell versus EC_{50} of 64 fmol/cell), less editing activity in the mouse liver when delivered with the same LNP formulation at the same 2.5-mg/kg dose (mean desired on-target editing of 26% versus 39%), and more unwanted bystander editing *in vivo* (2.8% versus 0.8%). Finally, we did not perform a long-term mouse study, although the precedent of the LNP treatment for the *PAH* c.842C>T (p.Pro281Leu) variant resulting in durable normalization of blood Phe levels in PKU mice, through 6 months of observation,[9] suggests that SpRY-ABE8.8/PAH4 LNP treatment would be similarly durable.

In conclusion, we demonstrate that a base editing strategy is effective in treating a humanized mouse model of the most common pathogenic variant found in individuals with the most common inborn error of metabolism. Future studies will focus on optimizing the base editing approach further and assessing its relative merits and demerits compared with a prime editing approach.

Data and code availability

The accession number for the next-generation sequencing data reported in this paper is Sequence Read Archive: PRJNA1026949. Other data are available in the Supplemental Data.

Supplemental information

Supplemental information can be found online at https://doi.org/10.1016/j.xhgg.2023.100253.

Acknowledgments

This work was supported by grants R35-HL145203, R01-HL148769, and U19-NS132301 from the US National Institutes of Health (K.M.); a Career Development Award from the American Heart Association (X.W.); a Graduate Research Fellowship from the US National Science Foundation (M.N.W.); the Children's Hospital of Philadelphia Research Institute Frontier Program (R.C.A.-N.); and the Winkelman Family Fund in Cardiovascular Innovation.

Author contributions

K.M., R.C.A.-N., M.-G.A., and X.W. supervised the work. D.L.B., M.N.W., P.Q., and X.W. contributed to wet laboratory experiments. K.M. performed bioinformatic analyses. H.S., G.D., and M.-G.A. performed mRNA production, LNP formulation, and cytokine/chemokine analyses. D.L.B., K.M., R.C.A.-N., and X.W. drafted the manuscript, and all authors contributed to the editing of the manuscript.

Declaration of interests

K.M. is an advisor to and holds equity in Verve Therapeutics and Variant Bio and is an advisor to LEXEO Therapeutics. R.C.A.-N. is an advisor to Latus Bio. M.-G.A. is a co-founder of and an advisor to AexeRNA Therapeutics. The University of Pennsylvania and Children's Hospital of Philadelphia have filed a patent application related to the use of base editing for the treatment of phenylketonuria (inventors D.L.B., K.M., R.C.A.-N., and X.W.).

Received: September 6, 2023
Accepted: October 19, 2023

References

1. Vockley, J., Andersson, H.C., Antshel, K.M., Braverman, N.E., Burton, B.K., Frazier, D.M., Mitchell, J., Smith, W.E., Thompson, B.H., Berry, S.A.; and American College of Medical Genetics and Genomics Therapeutics Committee (2014). Phenylalanine hydroxylase deficiency: diagnosis and management guideline. Genet. Med. *16*, 188–200. https://doi.org/10.1038/gim.2013.157.
2. Jurecki, E.R., Cederbaum, S., Kopesky, J., Perry, K., Rohr, F., Sanchez-Valle, A., Viau, K.S., Sheinin, M.Y., and Cohen-Pfeffer, J.L. (2017). Adherence to clinic recommendations among patients with phenylketonuria in the United States. Mol. Genet. Metab. *120*, 190–197. https://doi.org/10.1016/j.ymgme.2017.01.001.
3. Hillert, A., Anikster, Y., Belanger-Quintana, A., Burlina, A., Burton, B.K., Carducci, C., Chiesa, A.E., Christodoulou, J.,

Đorđević, M., Desviat, L.R., et al. (2020). The genetic landscape and epidemiology of phenylketonuria. Am. J. Hum. Genet. *107*, 234–250. https://doi.org/10.1016/j.ajhg.2020.06.006.

4. Brooks, D.L., Whittaker, M.N., Qu, P., Musunuru, K., Ahrens-Nicklas, R.C., and Wang, X. (2023). Efficient in vivo prime editing corrects the most frequent phenylketonuria variant, associated with high unmet medical need. Am. J. Hum. Genet. https://doi.org/10.1016/j.ajhg.2023.10.005.
5. Leuders, S., Wolfgart, E., Ott, T., du Moulin, M., van Teeffelen-Heithoff, A., Vogelpohl, L., Och, U., Marquardt, T., Weglage, J., Feldmann, R., and Rutsch, F. (2014). Influence of PAH genotype on sapropterin response in PKU: results of a single-center cohort study. JIMD Rep. *13*, 101–109. https://doi.org/10.1007/8904_2013_263.
6. Burton, B.K., Longo, N., Vockley, J., Grange, D.K., Harding, C.O., Decker, C., Li, M., Lau, K., Rosen, O., Larimore, K., et al. (2020). Pegvaliase for the treatment of phenylketonuria: results of the phase 2 dose-finding studies with long-term follow-up. Mol. Genet. Metab. *130*, 239–246. https://doi.org/10.1016/j.ymgme.2020.06.006.
7. Komor, A.C., Kim, Y.B., Packer, M.S., Zuris, J.A., and Liu, D.R. (2016). Programmable editing of a target base in genomic DNA without double-stranded DNA cleavage. Nature *533*, 420–424. https://doi.org/10.1038/nature17946.
8. Gaudelli, N.M., Komor, A.C., Rees, H.A., Packer, M.S., Badran, A.H., Bryson, D.I., and Liu, D.R. (2017). Programmable base editing of AT to GC in genomic DNA without DNA cleavage. Nature *551*, 464–471. https://doi.org/10.1038/nature24644.
9. Brooks, D.L., Carrasco, M.J., Qu, P., Peranteau, W.H., Ahrens-Nicklas, R.C., Musunuru, K., Alameh, M.-G., and Wang, X. (2023). Rapid and definitive treatment of phenylketonuria in variant-humanized mice with corrective editing. Nat. Commun. *14*, 3451. https://doi.org/10.1038/s41467-023-39246-2.
10. Desviat, L.R., Pérez, B., Gámez, A., Sánchez, A., García, M.J., Martínez-Pardo, M., Marchante, C., Bóveda, D., Baldellou, A., Arena, J., et al. (1999). Genetic and phenotypic aspects of phenylalanine hydroxylase deficiency in Spain: molecular survey by regions. Eur. J. Hum. Genet. *7*, 386–392. https://doi.org/10.1038/sj.ejhg.5200312.
11. Zhu, T., Ye, J., Han, L., Qiu, W., Zhang, H., Liang, L., and Gu, X. (2017). The predictive value of genetic analyses in the diagnosis of tetrahydrobiopterin (BH4)-responsiveness in Chinese phenylalanine hydroxylase deficiency patients. Sci. Rep. *7*, 6762. https://doi.org/10.1038/s41598-017-06462-y.
12. Chatterjee, P., Lee, J., Nip, L., Koseki, S.R.T., Tysinger, E., Sontheimer, E.J., Jacobson, J.M., and Jakimo, N. (2020). A Cas9 with PAM recognition for adenine dinucleotides. Nat. Commun. *11*, 2474. https://doi.org/10.1038/s41467-020-16117-8.
13. Walton, R.T., Christie, K.A., Whittaker, M.N., and Kleinstiver, B.P. (2020). Unconstrained genome targeting with near-PAMless engineered CRISPR-Cas9 variants. Science *368*, 290–296. https://doi.org/10.1126/science.aba8853.
14. Richter, M.F., Zhao, K.T., Eton, E., Lapinaite, A., Newby, G.A., Thuronyi, B.W., Wilson, C., Koblan, L.W., Zeng, J., Bauer, D.E., et al. (2020). Phage-assisted evolution of an adenine base editor with improved Cas domain compatibility and activity. Nat. Biotechnol. *38*, 883–891. https://doi.org/10.1038/s41587-020-0453-z.
15. Gaudelli, N.M., Lam, D.K., Rees, H.A., Solá-Esteves, N.M., Barrera, L.A., Born, D.A., Edwards, A., Gehrke, J.M., Lee, S.J., Liquori, A.J., et al. (2020). Directed evolution of adenine base editors with increased activity and therapeutic application. Nat. Biotechnol. *38*, 892–900. https://doi.org/10.1038/s41587-020-0491-6.
16. Ponnienselvan, K., Liu, P., Nyalile, T., Oikemus, S., Maitland, S.A., Lawson, N.D., Luban, J., and Wolfe, S.A. (2023). Reducing the inherent auto-inhibitory interaction within the pegRNA enhances prime editing efficiency. Nucleic Acids Res. *51*, 6966–6980. https://doi.org/10.1093/nar/gkad456.
17. Kingwell, K. (2022). Base editors hit the clinic. Nat. Rev. Drug Discov. *21*, 545–547. https://doi.org/10.1038/d41573-022-00124-z.
18. Kleinstiver, B.P., Prew, M.S., Tsai, S.Q., Topkar, V.V., Nguyen, N.T., Zheng, Z., Gonzales, A.P.W., Li, Z., Peterson, R.T., Yeh, J.R.J., et al. (2015). Engineered CRISPR-Cas9 nucleases with altered PAM specificities. Nature *523*, 481–485. https://doi.org/10.1038/nature14592.
19. Hu, J.H., Miller, S.M., Geurts, M.H., Tang, W., Chen, L., Sun, N., Zeina, C.M., Gao, X., Rees, H.A., Lin, Z., and Liu, D.R. (2018). Evolved Cas9 variants with broad PAM compatibility and high DNA specificity. Nature *556*, 57–63. https://doi.org/10.1038/nature26155.
20. Nishimasu, H., Shi, X., Ishiguro, S., Gao, L., Hirano, S., Okazaki, S., Noda, T., Abudayyeh, O.O., Gootenberg, J.S., Mori, H., et al. (2018). Engineered CRISPR-Cas9 nuclease with expanded targeting space. Science *361*, 1259–1262. https://doi.org/10.1126/science.aas9129.
21. Miller, S.M., Wang, T., Randolph, P.B., Arbab, M., Shen, M.W., Huang, T.P., Matuszek, Z., Newby, G.A., Rees, H.A., and Liu, D.R. (2020). Continuous evolution of SpCas9 variants compatible with non-G PAMs. Nat. Biotechnol. *38*, 471–481. https://doi.org/10.1038/s41587-020-0412-8.
22. Musunuru, K., Chadwick, A.C., Mizoguchi, T., Garcia, S.P., DeNizio, J.E., Reiss, C.W., Wang, K., Iyer, S., Dutta, C., Clendaniel, V., et al. (2021). In vivo CRISPR base editing of PCSK9 durably lowers cholesterol in primates. Nature *593*, 429–434. https://doi.org/10.1038/s41586-021-03534-y.

HGGA, Volume *5*

Supplemental information

A base editing strategy using mRNA-LNPs for *in vivo* correction of the most frequent phenylketonuria variant

Dominique L. Brooks, Madelynn N. Whittaker, Hooda Said, Garima Dwivedi, Ping Qu, Kiran Musunuru, Rebecca C. Ahrens-Nicklas, Mohamad-Gabriel Alameh, and Xiao Wang

Figure S1. On-target and Bystander Editing with Plasmid Transfections, Related to Figure 1B.

Results of next-generation sequencing, with the mean percentage for each nucleotide at each position indicated. Values ≤ 0.1% are not shown. Green = A, yellow = G, orange = C, purple = T. The red box indicates the c.1222C>T adenine; black boxes indicate sites of unwanted adenine bystander editing.

Reference		A	A	G	G	G	C	C	A	A	G	G	T	A	T	T	G	T	G	G	C	A	G	C
sgRNA																								
SpG-ABE7.10/PAH1	A	99.9	99.9						98.1	99.3				99.8								99.9		
	G			100	99.9	100			1.9	0.6	100	100		0.2			99.9		100	99.9			99.9	
	C						99.9	99.9													99.9			100
	T												99.9		100	99.9		99.9						
SpG-ABE8.20/PAH1	A	99.4	94.1						92.6	99				98.8								99.9		
	G	0.6	5.8	100	100	100	0.5	0.2	7.4	0.9	100	100		1.2			100		100	99.9			99.9	
	C						98.4	99.7													99.9			100
	T						1.0						99.8		100	99.9		99.9						
SpG-ABE8e/PAH1	A	99.6	97.9						92.4	99				98.9								99.9		
	G	0.3	2.1	100	99.9	100			7.6	0.9	100	100		1.1			100		99.9	99.9			99.9	
	C						99.6	99.9													99.9			100
	T						0.2						99.9		99.9	100		99.9						
SpRY-ABE8e/PAH1	A	99.8	98.3						95	99.1				98.7								99.9		
	G	0.2	1.6	100	100	100			5.0	0.8	100	100		1.3			100		100	100			99.9	
	C						99.6	99.8													99.9			100
	T						0.3						99.9		99.9	99.9		99.9						
SpRY-ABE8.8/PAH1	A	99.9	99.7						98.3	99.6				99.7								99.9		
	G		0.2	100	99.9	99.9			1.6	0.4	100	100		0.2			100		100	99.9			99.9	
	C						99.9	100													99.9			100
	T												99.9		99.9	99.9		99.9						
SpRY-ABE8e/PAH2	A	99.8	99.9						94.2	96.6				98.4								99.9		
	G			100	100	99.9		0.2	5.7	3.3	100	100		1.5			100		100	100			99.9	
	C						99.8	99.7													99.9			100
	T						0.2						99.9		100	99.9		99.9						
SpRY-ABE8.8/PAH2	A	100	99.9						96.7	99.1				99.7								99.9		
	G			100	99.9	100			3.3	0.8	100	100		0.3			100		100	100			100	
	C						99.9	99.9													99.9			100
	T												99.9		100	100		99.9						
iSpyMac-ABE8.8/PAH3	A	99.9	99.9						90.8	97.3				99.7								99.9		
	G			100	99.9	100			9.1	2.6	100	100		0.3			100		100	99.9			99.9	
	C						99.9	99.9													99.9			100
	T												99.9		100	100		99.9						
SpRY-ABE8e/PAH3	A	99.9	99.9						94.1	95.9				98.5								99.9		
	G			99.9	100	99.9		0.2	5.9	4.1	100	100		1.4			100		99.9	100			99.9	
	C						99.8	99.6													99.9			100
	T												99.9		100	100		99.9						
SpRY-ABE8.8/PAH3	A	99.9	99.9						95.7	98.5				99.8								99.9		
	G			100	99.9	99.9			4.2	1.4	100	100		0.2			100		100	99.9			99.9	
	C						99.8	99.9													99.9			100
	T												99.9		100	100		99.9						
iSpyMac-ABE8.8/PAH4	A	99.9	99.9						93.3	96.8				99.2								99.9		
	G			100	99.9	99.9			6.6	3.1	100	100		0.8			100		99.9	100			99.9	
	C						99.9	99.9													99.9			100
	T												99.9		100	99.9		99.9						
SpRY-ABE8e/PAH4	A	99.9	99.9					0.2	93.7	94.4				96.6								99.9		
	G			100	99.9	100		0.2	6.3	5.6	100	100		3.4			100		99.9	99.9			99.9	
	C						99.9	99.6													99.9			100
	T												99.9		99.9	99.9		99.9						
SpRY-ABE8.8/PAH4	A	100	99.9						86.7	93.6				99								99.9		
	G			100	99.9	100			13.3	6.3	100	100		0.9			100		99.9	99.9			100	
	C						99.8	99.7													99.9			100
	T												99.9		100	99.9		99.9						
SpRY-ABE8e/PAH5	A	99.9	99.9						90.7	91				91.9								99.7		
	G			100	99.9	99.9			9.2	8.9	100	100		8.1			100		100	100		0.2	99.9	
	C						99.9	99.9													99.9			100
	T												99.9		100	99.9		100						
SpRY-ABE8.8/PAH5	A	99.9	99.9						90.3	91.7				96.7								99.9		
	G			99.9	99.9	99.9			9.7	8.2	100	100		3.2			100		99.9	99.9			99.9	
	C						99.9	99.9													99.9			100
	T												99.9		99.9	99.9		99.9						
ABE8.8/PAH5	A	99.9	99.9						99.0	99.5				99.7								99.9		
	G			100	99.9	99.9			1.0	0.4	100	100		0.3			100		100	100			100	
	C						99.9	100													99.9			100
	T												99.9		99.9	100		99.9						
SpG-ABE7.10/PAH6	A	99.9	99.9						98.8	98.7				93.1								99.9		
	G			100	99.9	100			1.2	1.3	100	100		6.9			100		100	99.9			99.9	
	C						99.9	100													100			100
	T												99.9		100	99.9		99.9						
SpG-ABE8.20/PAH6	A	99.9	99.9						83.2	84.2				80.9								99.8		
	G			100	99.9	99.9			16.7	15.8	100	100		19.1			100		100	99.9			99.9	
	C						99.9	99.9													99.9			100
	T												99.8		99.9	100		99.9						
SpG-ABE8e/PAH6	A	99.9	99.9						94.3	94.3				94.1								99.7		
	G			99.9	99.9	100			5.6	5.6	100	100		5.9			100		99.9	99.9		0.3	100	
	C						99.9	99.9													99.9			100
	T												99.9		99.9	99.9		99.8						
SpRY-ABE8e/PAH6	A	99.9	99.9						93.6	93.7				93.3								99.6		
	G			100	99.9	100			6.4	6.3	100	100		6.7			100		100	99.9		0.4	100	
	C						99.9	100													99.9			100
	T												99.9		99.9	99.9		99.9						
SpRY-ABE8.8/PAH6	A	99.9	99.9						95.2	93.7				90.3								99.9		
	G			100	99.9	100			4.7	6.2	100	100		9.6			100		100	100			100	
	C						99.9	100													99.9			100
	T												99.9		99.9	99.9		99.9						

Figure S2. On-target and Bystander Editing with LNP Transfections, Related to Figure 1C.

Results of next-generation sequencing, with the mean percentage for each nucleotide at each position indicated. Values ≤ 0.1% are not shown. Green = A, yellow = G, orange = C, purple = T. The red box indicates the c.1222C>T adenine; black boxes indicate sites of unwanted adenine bystander editing.

sgRNA

Reference		A	A	G	G	G	C	C	A	A	G	G	T	A	T	T	G	T	G	G	C	A	G	C
39 fg/cell SpRY-ABE8.8/PAH4	A	99.9	99.9						99.2	99.8				99.8								99.9		
	G			100	99.9	99.9			0.8	0.2	100	100					99.9		100	100			100	
	C						99.9	99.9													100			100
	T	99.9	99.9						99.2	99.8				99.8								99.9		
78 fg/cell SpRY-ABE8.8/PAH4	A	99.9	99.9						98.5	99.5				99.8								99.9		
	G			99.9	99.9	99.9			1.5	0.4	100	99.9		0.2			100		100	100			99.9	
	C						99.9	100													100			100
	T												99.8		100	100		99.9						
156 fg/cell SpRY-ABE8.8/PAH4	A	99.9	99.9						94.3	98.7				99.4								99.9		
	G			99.9	99.9	99.9			5.7	1.2	100	100		0.6			99.9		100	100			100	
	C						99.9	100													100			100
	T												99.8		100	100		99.9						
313 fg/cell SpRY-ABE8.8/PAH4	A	99.9	99.9						86	96.6				98.5								99.9		
	G			100	99.9	99.9			13.9	3.3	99.9	100		1.5			99.9		100	99.9			99.9	
	C						99.9	100													100			100
	T												99.8		99.9	99.9		99.9						
625 fg/cell SpRY-ABE8.8/PAH4	A	99.9	99.8						69.3	92.4				97.0								99.8		
	G			100	99.9	99.9			30.7	7.5	99.9	100		3.0			100		99.9	99.9			99.9	
	C						99.9	99.9													99.9			99.9
	T												99.8		100	100		99.9						
1250 fg/cell SpRY-ABE8.8/PAH4	A	99.9	99.9						49.1	86.5				93.8								99.9		
	G			99.9	99.9	99.9			50.9	13.4	99.9	100		6.2			99.9		99.9	99.9			99.9	
	C						99.8	99.9													99.9			99.9
	T												99.7		100	100		99.8						
2500 fg/cell SpRY-ABE8.8/PAH4	A	99.9	99.9					0.3	26.6	77.3				88.5								99.8		
	G			99.9	99.9	99.9			73.4	22.6	99.9	100		11.5			99.9		100	100			99.9	
	C						99.9	99.6													99.8			100
	T												99.7		100	99.9		99.8						
5000 fg/cell SpRY-ABE8.8/PAH4	A	99.9	99.9						27.7	78.8				90								99.9		
	G			100	99.9	99.9			72.2	21.1	99.9	100		9.9			99.9		99.9	99.9			99.9	
	C						99.9	99.9													99.9			100
	T												99.8		100	100		99.9						
10000 fg/cell SpRY-ABE8.8/PAH4	A	99.9	100				0.2		28.5	79			0.2	91.4								100		
	G			99.9	99.9	99.9			71.4	20.7	100	100		8.6			100		99.9	99.9			100	
	C						99.8	99.8					0.2								99.9			100
	T									0.2			99.4		100	99.9		99.9						

Figure S3. Phenylketonuria Phenotypes in Humanized R408W Mice.

(A) Sanger sequencing chromatograms showing the generation of a humanized mouse model via Cas9-mediated homology-directed repair in mouse zygotes. At the top is sequence from a wild-type C57BL/6J mouse. At the bottom is sequence from a mouse homozygous for the humanized *Pah* c.1222C>T allele. The red arrow indicates the site of the c.1222C>T variant, and the black arrows indicate the sites of additional changes that humanize the local region of the mouse *Pah* gene.
(B) Homozygous R408W mice with PKU as evidenced by mild hypopigmentation of the fur, on left, compared to heterozygous R408W non-PKU littermates, on right.
(C) Blood phenylalanine levels and weights of homozygous R408W PKU (n = 5 animals) and heterozygous R408W non-PKU (n = 3 animals) age-matched (6 weeks of age) littermates. Lines = mean values. P values calculated with unpaired t-test.

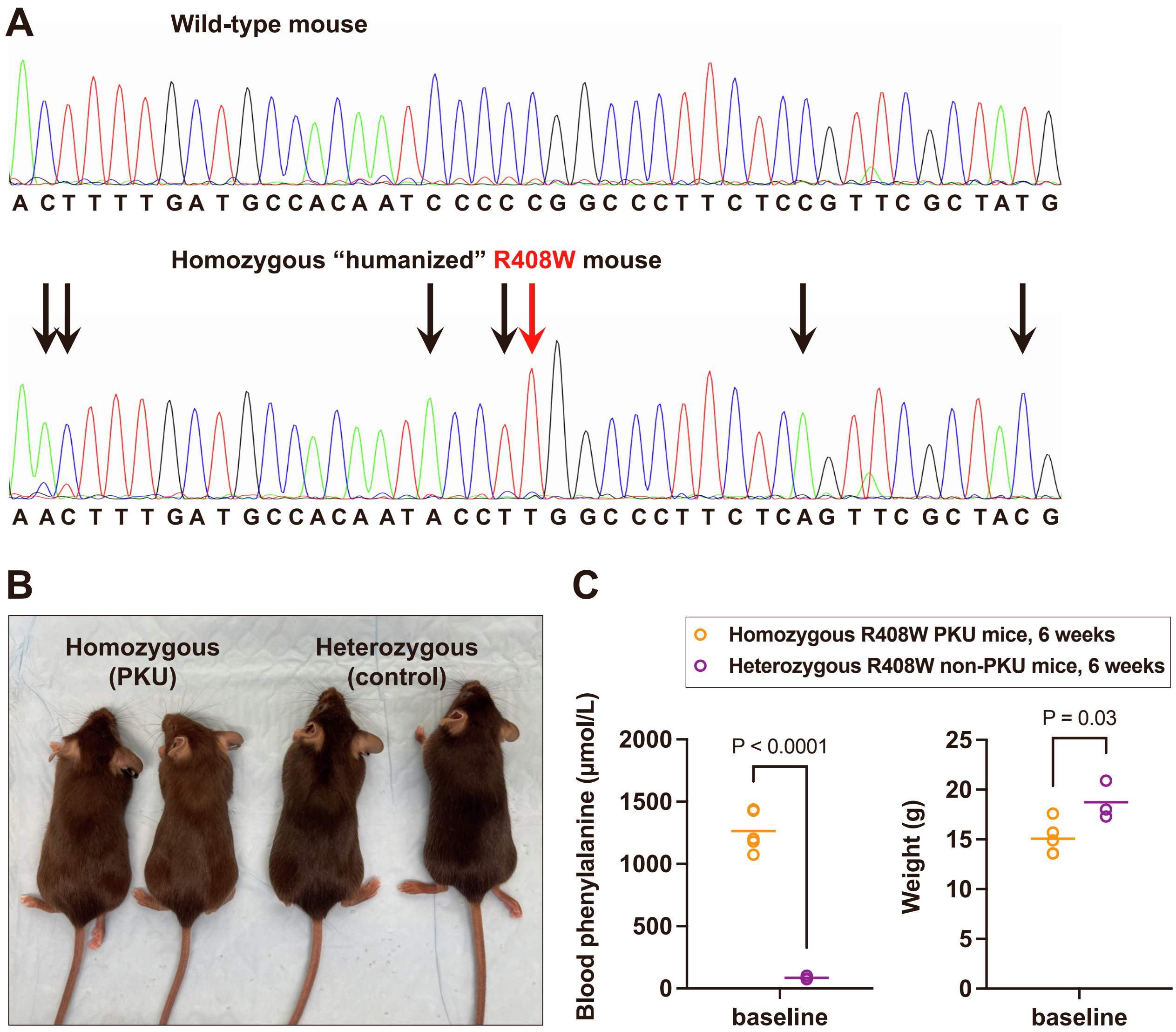

Figure S4. Assessment of Mouse Liver Following LNP Treatment or Vehicle Treatment.

(A) Blood aspartate aminotransferase (AST) levels in homozygous PKU mice following treatment with 5 mg/kg dose of SpRY-ABE8.8/PAH4 LNPs (n = 3 animals) or with 2.5 mg/kg dose of LNPs (n = 3 animals), comparing levels at various timepoints up to 7 days following treatment to levels in vehicle-treated homozygous PKU control (n = 4 animals) and vehicle-treated heterozygous non-PKU control (n = 4 animals) age-matched (approximately 8 weeks of age) colonymates (1 blood sample per timepoint).
(B) Blood alanine aminotransferase (ALT) levels in the LNP-treated or vehicle-treated PKU or non-PKU mice shown in (A).
(C) Representative liver histology (hematoxylin/eosin staining) at 20× magnification upon necropsy at 1 week after LNP treatment of PKU mice or vehicle treatment of a non-PKU mouse shown in (A) and (B).
Lines in pictures = distance of 50 μm.
Lines in graphs = mean values.

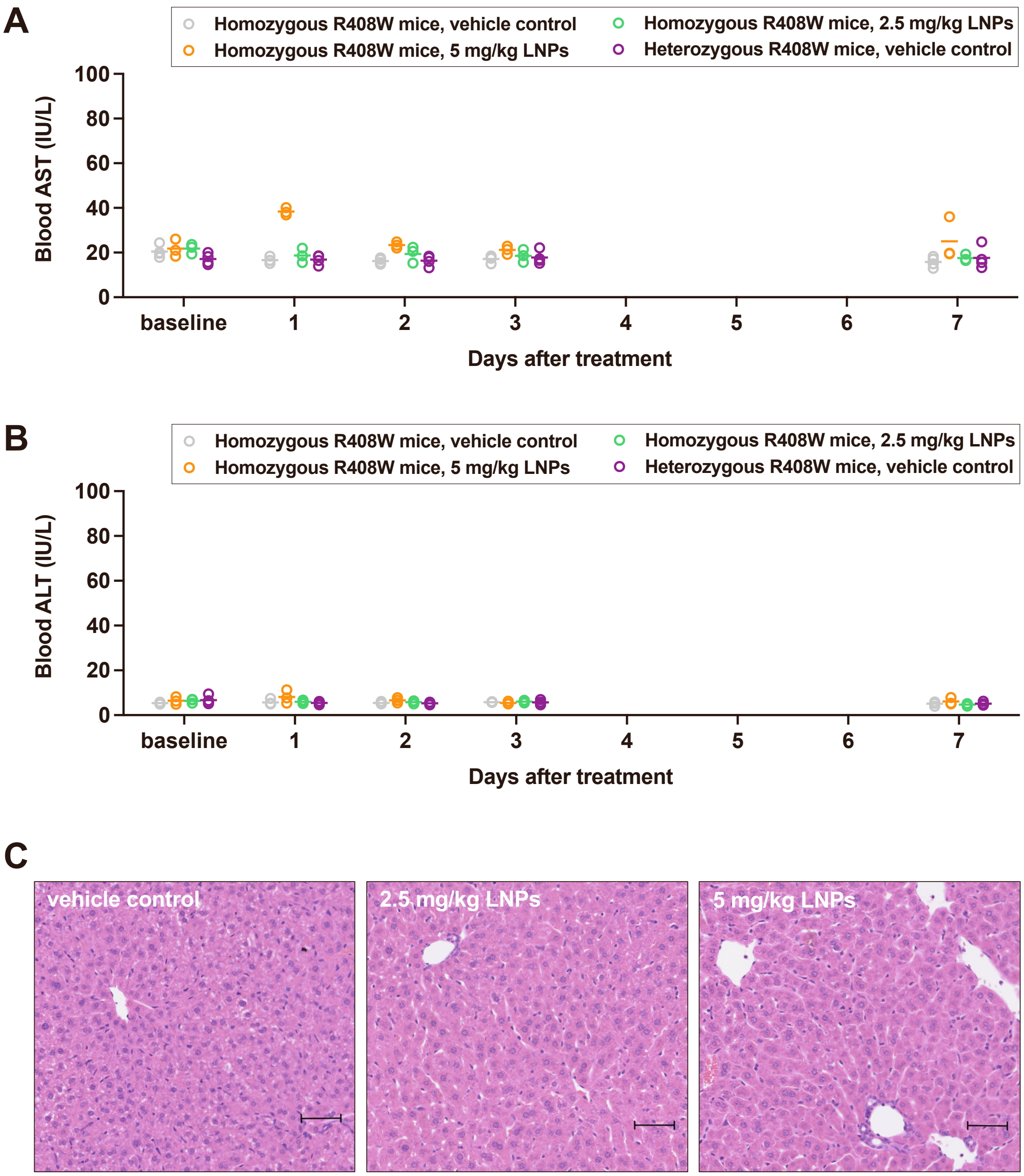

Figure S5. Prime Editing to Correct *PAH* c.1222C>T Variant in Humanized Mice.

(A) Blood phenylalanine levels in homozygous PKU mice following treatment with PEmax/P56/N19 LNPs (n = 3 animals) at various timepoints up to 4 days following treatment (1 blood sample per timepoint). Lines = mean values.
(B) Corrective *PAH* c.1222C>T editing (determined from genomic DNA) in each of 8 liver samples (two samples each from the four lobes) collected from each treated mouse upon necropsy 1 week after treatment, calculated as the proportion of aligned sequencing reads in which the c.1222C>T adenine variant is edited to guanine, irrespective of other edits.

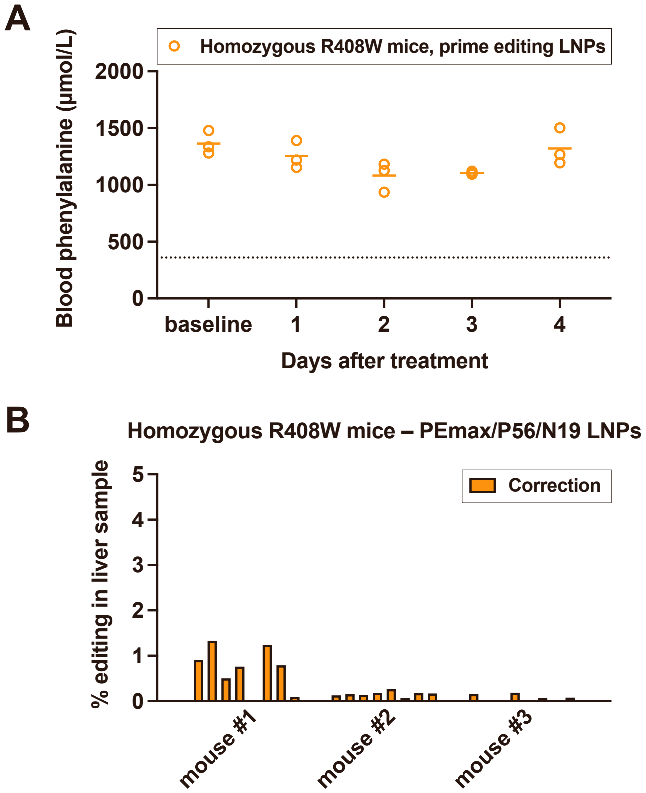

Table S1. Cytokine and Chemokine Levels in R408W Humanized Mice Following LNP Treatment or Vehicle Treatment.

Time	IFN-γ	CXCL-1	TNF-α	MCP-1	IL-12p70	RANTES	IL-1β	IP-10	GM-CSF	IL-10	IFN-β	IFN-α	IL-6
**Homozygous R408W PKU mouse, 8 weeks of age, 2.5 mg/kg SpRY-ABE8.8/PAH4 LNPs**													
**baseline**	3.40	29.17	9.40	11.59	1.52	33.83	1.43	231.12	4.35	11.01	0.00	3.41	7.86
**4 hours**	4.45	109.57	17.59	115.68	1.73	31.87	13.04	2374.70	2.60	3.49	0.00	10.27	1675.05
**24 hours**	3.40	24.64	6.06	7.73	1.62	32.43	1.25	296.92	1.64	0.44	0.00	3.41	32.90
**Homozygous R408W PKU mouse, 8 weeks of age, vehicle control**													
**baseline**	2.20	15.97	7.69	4.97	1.20	30.19	2.72	135.86	2.50	0.73	0.00	2.50	5.75
**4 hours**	2.65	18.12	5.04	13.43	1.66	24.09	3.73	90.19	1.34	0.44	0.00	2.23	289.85
**24 hours**	1.59	25.62	8.25	6.75	1.26	16.01	6.62	128.72	0.93	1.46	0.00	1.39	298.85
**Heterozygous R408W non-PKU mouse, 8 weeks of age, vehicle control**													
**baseline**	1.89	8.55	4.08	9.58	1.14	20.81	0.29	66.45	3.49	4.74	0.00	1.73	2.12
**4 hours**	2.35	43.47	5.04	16.40	1.87	21.90	4.55	109.61	2.14	3.49	0.00	2.50	198.09
**24 hours**	1.89	27.58	6.06	8.76	1.35	27.40	8.55	142.82	2.88	4.74	0.00	2.67	67.20

All cytokine/chemokine measurements are in units of pg/mL.

Table S2. Assessment of Off-target Editing in *PAH* c.1222C>T Homozygous HuH-7 Cells.

Genomic site	Protospacer/PAM sequence[a]	Amplicon (chromosome: position range)[b]	Treated sample 1[c]	Treated sample 2	Treated sample 3	Control sample 1	Control sample 2	Control sample 3
PAH	GGCCAAGGTATTGTGGCAGCAAA	chr12:102840425+102840539	75.87	78.73	77.76	0.20	0.16	0.20
OT1	GGCCAAGGgATTGTGG-AGCTGT	chr1:67956357-67956526	0.08	0.06	0.09	0.10	0.06	0.07
OT2	GGCCAAGcTATTGCTGGCAGCCAG	chr1:165405262+165405443	0.06	0.06	0.04	0.04	0.04	0.03
OT3	GGCCAAGGT-TTcTGGCAGCTGG	chr1:182646888+182647080	0.06	0.06	0.08	0.04	0.04	0.03
OT4	GcCCAAGGTATTGTGGCA-CCGC	chr12:98762684-98762861	0.07	0.07	0.07	0.08	0.06	0.05
OT5	tGCCAAGGTATTGT-GCAGCCAG	chr13:56216949+56217104	0.09	0.05	0.13	0.13	0.06	0.04
OT6	GGCCAAGG-AaTGTGGCAGCTTC	chr13:102535487-102535594	0.11	0.08	0.10	0.09	0.11	0.08
OT7	GGCC-AGGcATTGTGGCAGCACC	chr13:112399414-112399538	0.06	0.06	0.04	0.04	0.09	0.06
OT8	GGCCAAGGT-TTGTGGCAaCCCT	chr14:98620044+98620168	0.05	0.07	0.04	0.02	0.04	0.04
OT9	GG-CAAGGTgTTGTGGCAGCTTT	chr17:79400839-79400958	0.03	0.03	0.03	0.03	0.05	0.04
OT10	GGCCAAGG-ATTGTGtCAGCCCA	chr5:156096522-156096673	0.04	0.09	0.06	0.05	0.08	0.06
OT11	GGCC-AGGTtTTGTGGCAGCATC	chr5:168457657-168457813	0.03	0.04	0.04	0.04	n.d.	0.03
OT12	aGCCAAGG-ATTGTGGCAGCTAC	chrX:105510661+105510860	0.18	0.20	0.11	0.06	0.06	0.05
OT13	GGCCAAGGTA--GTGGCAGaGCA	chr1:39317216+39317406	0.06	0.05	0.06	0.06	0.03	0.05
OT14	GGCCA--GTATgGTGGCAGCAGA	chr1:91658136-91658280	0.05	0.06	0.02	0.05	0.04	0.05
OT15	GGCCAAGGTAT--TGGCAGgTAT	chr1:143710105-143710246	0.09	0.09	0.08	0.08	0.07	0.11
OT16	GtCCAAGGTA--GTGGCAGCAAA	chr1:184534886+184535019	0.06	0.05	0.05	0.07	0.08	0.04
OT17	GGCCAAGGTg--GTGGCAGCCAC	chr11:16790220+16790462	0.06	0.01	0.03	0.02	0.04	0.04
OT18	GG--AAGaTATTGTGGCAGCCGT	chr11:42929612-42929802	0.09	0.12	0.07	0.08	0.07	0.09
OT19	GG--AAGGTATTGTtGCAGCTGT	chr11:43718128+43718289	0.06	0.11	0.10	0.07	0.11	0.07
OT20	GcCCAAGGTATTGTGGCACCGCAGA	chr12:98762684-98762861	0.09	0.04	0.07	0.07	0.03	0.05
OT21	GGC--AGGTATTGTtGCAGCCCC	chr13:95505215+95505411	0.10	0.04	0.04	0.07	0.07	0.04
OT22	GGCCAAG--ATTGTGtCAGCTAG	chr15:73613514+73613688	0.07	0.05	0.09	0.06	0.05	0.09
OT23	GG--AAGGTATTcTGGCAGCTCT	chr16:1606113-1606257	0.02	0.09	0.04	0.06	0.04	0.07
OT24	GGCCAAaGTATTGTGG--GCTAA	chr16:65767602-65767791	0.04	0.05	0.03	0.05	0.05	0.05
OT25	GG--gAGGTATTGTGGCAGCTCC	chr17:50567554-50567721	n.d.	n.d.	n.d.	n.d.	n.d.	n.d.
OT26	GGCCAAGGTAgTGTG--AGCTGA	chr18:36992979+36993082	n.d.	n.d.	n.d.	n.d.	n.d.	n.d.
OT27	GGCCAgG--ATTGTGGCAGCAGA	chr2:21132399+21132532	0.13	0.07	0.05	0.04	0.06	0.04
OT28	G--CAAGGTATTGTGGCtGCCAC	chr2:65797812+65797991	0.07	0.07	n.d.	0.06	0.06	0.04
OT29	GGCCAAGG--TTGTaGCAGCTTG	chr2:74493829-74493998	0.02	0.04	0.03	0.03	0.03	0.05
OT30	GGCCAA--TATTGaGGCAGCTAT	chr2:75098514-75098650	0.05	0.07	0.06	0.03	0.07	0.04
OT31	GGCCAAGGTAgTGTGG--GCAGA	chr2:170268241+170268437	0.10	0.09	0.10	0.07	0.09	0.10
OT32	GGCCACCAGGaATTGTGGCAGCCAA	chr20:49750986+49751159	0.03	0.03	0.02	0.02	0.05	0.04
OT33	GGCCAAGG--cTGTGGCAGCTTC	chr20:57441083-57441206	0.01	0.03	0.01	0.01	0.01	0.02
OT34	GGCCAAGGTtTTGT--CAGCCAC	chr3:3727491-3727618	0.05	0.06	0.06	0.04	0.03	0.05
OT35	GGCCAAGaTATTGTGGC--CAAT	chr4:33129101-33129296	0.09	n.d.	0.09	0.08	0.07	0.11
OT36	GGCCAAGGgATTGTG--AGCAGA	chr4:176650261-176650399	0.08	0.07	0.04	0.04	0.06	0.06
OT37	GcCCAAGG--TTGTGGCAGCCCA	chr5:15842116+15842313	n.d.	n.d.	n.d.	n.d.	n.d.	n.d.
OT38	GtCCAAGGTATTGT--CAGCTTG	chr5:42644125-42644285	0.07	0.06	0.07	0.12	0.09	0.06
OT39	GGCCAAGGTgT--TGGCAGCTGC	chr5:138315728+138315864	0.05	0.03	0.05	0.03	0.07	0.04
OT40	GGCCAAGGTATTGTG--AGgGGA	chr6:23532313+23532467	0.07	0.06	0.05	0.10	0.07	0.08
OT41	GG--AAtGTATTGTGGCAGCTCC	chr6:36145377-36145550	0.06	0.06	0.07	0.05	0.07	0.05
OT42	GGCCAAGGTA--GTGGCAaCAGA	chr6:50411635+50411817	0.08	0.05	0.08	0.06	0.09	0.08
OT43	GtCCAAGGTAT--TGGCAGCTTC	chr6:68856951+68857064	0.06	0.08	0.06	0.08	0.08	0.07
OT44	GGCCAAGGTAT--TGGCAGgGAT	chr6:115167457+115167613	0.07	0.04	0.08	0.11	0.08	0.05
OT45	GGCCtAGGTATTGT--CAGCTCT	chr6:123648344+123648515	n.d.	n.d.	n.d.	n.d.	n.d.	n.d.
OT46	GGCCAAGGTA--GTGGgAGCTTT	chr7:11182970-11183157	0.07	0.09	0.06	0.09	0.05	0.07
OT47	GGCCAAGGTA--GTGGCAGtAAG	chr8:10159022+10159160	0.05	0.08	0.08	0.10	0.07	0.06
OT48	GG--AAGGTATTaTGGCAGCAGC	chr8:80885442+80885634	0.07	0.06	0.08	0.07	0.10	0.07
OT49	GGC--AGGTATTGTGGCAGaAGT	chr8:122080739-122080887	0.07	0.05	0.06	0.06	0.05	0.05
OT50	GcCCAAGGTAT--TGGCAGCTTT	chrX:146875655+146875796	n.d.	n.d.	n.d.	n.d.	n.d.	n.d.
OT51	aGCCAcGGTATTGTGGCAGCTTG	chr1:160709019+160709185	0.03	n.d.	0.03	0.06	0.02	0.07
OT52	GGaCAAGGTATTGTGGgAGCCCA	chr10:58773438+58773613	0.05	n.d.	0.09	0.08	0.11	n.d.
OT53	GGCCAAGGTAcTGTGtCAGCCAG	chr12:51752733-51752923	0.10	n.d.	0.07	0.08	0.13	0.14
OT54	GGCCAAGGaATTcTGGCAGCCAC	chr14:78442315+78442449	0.06	n.d.	0.08	0.07	0.10	0.09
OT55	GGCCAAGGTgaTGTGGCAGCATG	chr15:62831968-62832156	0.07	n.d.	0.10	0.06	0.07	0.09
OT56	GGCCAAGGTATgGTGcCAGCTTG	chr2:118901813-118901955	0.04	0.06	0.05	0.08	0.05	0.08
OT57	GGCCAAGGgtTTGTGGCAGCACA	chr8:47691583-47691764	0.08	0.04	0.08	0.05	0.06	0.07

[a]The *PAH* on-target sequence and top in-silico-nominated candidate off-target sequences with mismatched positions and bulges indicated by lower case letters and dashes.

[b]Coordinates in the GRCh38/hg38 assembly of the human genome; "+" and "-" indicate forward or reverse orientation of protospacer/PAM sequence.

[c]n.d. = PCR was unsuccessful for the genomic site.

Table S3. PCR Primers for Next-generation Sequencing.

Site	Forward primer	Reverse primer
PAH (human)	TCGTCGGCAGCGTCAGATGTGTATAAGAGACAG TGTCCAAGACCTCAATCCTTTG	GTCTCGTGGGCTCGGAGATGTGTATAAGAGACAG CACTCAAGCCTGTGGTTTTG
PAH (mouse)	TCGTCGGCAGCGTCAGATGTGTATAAGAGACAG GGCTCACAGCAAGGAAAAAG	GTCTCGTGGGCTCGGAGATGTGTATAAGAGACAG AGGTAGTCCCAGCAGGTGTG
PAH (OT analysis)	TCGTCGGCAGCGTCAGATGTGTATAAGAGACAG AAGCTGCTGGGTATTGTCCA	GTCTCGTGGGCTCGGAGATGTGTATAAGAGACAG TCAAGCCTGTGGTTTTGGTC
OT1	TCGTCGGCAGCGTCAGATGTGTATAAGAGACAG GGTTTCAAATCAGTCTGGCCA	GTCTCGTGGGCTCGGAGATGTGTATAAGAGACAG CCTGCCCTTCAAAACTAGTGG
OT2	TCGTCGGCAGCGTCAGATGTGTATAAGAGACAG TCCAACTCAGGGCTTCAATTAA	GTCTCGTGGGCTCGGAGATGTGTATAAGAGACAG TGTATCCTGTTGTATGCCGAAG
OT3	TCGTCGGCAGCGTCAGATGTGTATAAGAGACAG GGCTTCTCGGGTCTGGAA	GTCTCGTGGGCTCGGAGATGTGTATAAGAGACAG TGCTGTGATCAAAAGGGATGA
OT4	TCGTCGGCAGCGTCAGATGTGTATAAGAGACAG GAGATGCAAAGCCCAGTGAG	GTCTCGTGGGCTCGGAGATGTGTATAAGAGACAG TCCTTTCCTGTCCTCCCAAC
OT5	TCGTCGGCAGCGTCAGATGTGTATAAGAGACAG CGACTTTGCACCTTCCTG	GTCTCGTGGGCTCGGAGATGTGTATAAGAGACAG AAGTGGACTCTCATGCAGGT
OT6	TCGTCGGCAGCGTCAGATGTGTATAAGAGACAG GGGGATGTGGCAGTAGAAGT	GTCTCGTGGGCTCGGAGATGTGTATAAGAGACAG TCTCCCTTTCCCAGCTTCTG
OT7	TCGTCGGCAGCGTCAGATGTGTATAAGAGACAG CTGGCTCTCTCTGCACCTG	GTCTCGTGGGCTCGGAGATGTGTATAAGAGACAG GAAATGTCTCATGGGCAGGC
OT8	TCGTCGGCAGCGTCAGATGTGTATAAGAGACAG GGCACTAATCCATTCATGAAGGT	GTCTCGTGGGCTCGGAGATGTGTATAAGAGACAG TGGCCCAGATAGAGTTGCTC
OT9	TCGTCGGCAGCGTCAGATGTGTATAAGAGACAG CACAGGGATGCTAGCTAGGC	GTCTCGTGGGCTCGGAGATGTGTATAAGAGACAG CAAAACCGAAGTCCCCACAC
OT10	TCGTCGGCAGCGTCAGATGTGTATAAGAGACAG TGCTTGCTTAGAAAATCCAGGG	GTCTCGTGGGCTCGGAGATGTGTATAAGAGACAG TCATTGCCCCATCTGGTGAA
OT11	TCGTCGGCAGCGTCAGATGTGTATAAGAGACAG CAGTGACCAGAGCTTCCATG	GTCTCGTGGGCTCGGAGATGTGTATAAGAGACAG CCACAGCCAAAATCACACCA
OT12	TCGTCGGCAGCGTCAGATGTGTATAAGAGACAG AGGCAGAAGAATAGGTCAGAGT	GTCTCGTGGGCTCGGAGATGTGTATAAGAGACAG CTGGGGCGAAAATCAAGTGT
OT13	TCGTCGGCAGCGTCAGATGTGTATAAGAGACAG CTGGCTTAGTGATGTGAAGGAC	GTCTCGTGGGCTCGGAGATGTGTATAAGAGACAG GAATCTCGAGCTGGGCAATG
OT14	TCGTCGGCAGCGTCAGATGTGTATAAGAGACAG AGGATCACTCTGGCTACTATGT	GTCTCGTGGGCTCGGAGATGTGTATAAGAGACAG TCCTTTGAATCTGACCCTTTCTT
OT15	TCGTCGGCAGCGTCAGATGTGTATAAGAGACAG TGCGTCAGGACAGAAAGTTTG	GTCTCGTGGGCTCGGAGATGTGTATAAGAGACAG TGCTACATAAAACGAAACCACCA
OT16	TCGTCGGCAGCGTCAGATGTGTATAAGAGACAG GGCAGAGAGTTCAGAAGGGT	GTCTCGTGGGCTCGGAGATGTGTATAAGAGACAG AGCACCCAAAGACCTCAAGT
OT17	TCGTCGGCAGCGTCAGATGTGTATAAGAGACAG AGAAGGACACTCAACTGGCA	GTCTCGTGGGCTCGGAGATGTGTATAAGAGACAG GAAGACAATCCCAGTGCTCT
OT18	TCGTCGGCAGCGTCAGATGTGTATAAGAGACAG GGGAACTTCACCACTGTTGAG	GTCTCGTGGGCTCGGAGATGTGTATAAGAGACAG GCTCTGCTTTTCCCTCCAG
OT19	TCGTCGGCAGCGTCAGATGTGTATAAGAGACAG AAAGCAAGCCACAGGACCAG	GTCTCGTGGGCTCGGAGATGTGTATAAGAGACAG TGATATGTTCAGAGGGCAGACT
OT20	TCGTCGGCAGCGTCAGATGTGTATAAGAGACAG GAGATGCAAAGCCCAGTGAG	GTCTCGTGGGCTCGGAGATGTGTATAAGAGACAG TCCTTTCCTGTCCTCCCAAC
OT21	TCGTCGGCAGCGTCAGATGTGTATAAGAGACAG AGGTTCTGAGCGTCCACA	GTCTCGTGGGCTCGGAGATGTGTATAAGAGACAG CCCCATGTGCCCATTGTAAG
OT22	TCGTCGGCAGCGTCAGATGTGTATAAGAGACAG GTTCTCCACTTAGTAATTTCAGA	GTCTCGTGGGCTCGGAGATGTGTATAAGAGACAG GTCCAGCATTTTGTTACCGC
OT23	TCGTCGGCAGCGTCAGATGTGTATAAGAGACAG ATCTTCCTCTGTCAGTGCCC	GTCTCGTGGGCTCGGAGATGTGTATAAGAGACAG CCCTGCTGACACCTGATTTT
OT24	TCGTCGGCAGCGTCAGATGTGTATAAGAGACAG CCCCACATGCTCCATCAGAA	GTCTCGTGGGCTCGGAGATGTGTATAAGAGACAG TGGGGCAAAAGCGATCATTT
OT25	TCGTCGGCAGCGTCAGATGTGTATAAGAGACAG GAGAGCAGGAGATTGGGGAA	GTCTCGTGGGCTCGGAGATGTGTATAAGAGACAG AGCAAGATTCCACCTGCTCT
OT26	TCGTCGGCAGCGTCAGATGTGTATAAGAGACAG CACCTGTAGTCCCAGCTCC	GTCTCGTGGGCTCGGAGATGTGTATAAGAGACAG CCAGGTTGAAGTGCAGTGTT

OT27	TCGTCGGCAGCGTCAGATGTGTATAAGAGACAG GATGGGGTGGAGTTAAGGCT	GTCTCGTGGGCTCGGAGATGTGTATAAGAGACAG TGGGTTCATCTCCAGAGCAT
OT28	TCGTCGGCAGCGTCAGATGTGTATAAGAGACAG TTAGGATCTCAGTGGGTGGG	GTCTCGTGGGCTCGGAGATGTGTATAAGAGACAG AGAGCAACTTTCATCCCTGC
OT29	TCGTCGGCAGCGTCAGATGTGTATAAGAGACAG ACATGGGGAAAGGCTAATTACC	GTCTCGTGGGCTCGGAGATGTGTATAAGAGACAG AAGTCCCTTACCAGCTCCTG
OT30	TCGTCGGCAGCGTCAGATGTGTATAAGAGACAG TAGGGATGTGGGTTTCAGGG	GTCTCGTGGGCTCGGAGATGTGTATAAGAGACAG TGAGAAAGATGAAGAGAGACCT
OT31	TCGTCGGCAGCGTCAGATGTGTATAAGAGACAG TCTGGAGAAAAGGGCAAACTC	GTCTCGTGGGCTCGGAGATGTGTATAAGAGACAG CACTCTGGCCTATAGATTTTACA
OT32	TCGTCGGCAGCGTCAGATGTGTATAAGAGACAG ACATGAAACACAAGGGCAGG	GTCTCGTGGGCTCGGAGATGTGTATAAGAGACAG ATGGGCTCAGGGAAGAAGAC
OT33	TCGTCGGCAGCGTCAGATGTGTATAAGAGACAG CATGCCAGGCCTCTCACC	GTCTCGTGGGCTCGGAGATGTGTATAAGAGACAG GAATCCCTGTCCCACCCTG
OT34	TCGTCGGCAGCGTCAGATGTGTATAAGAGACAG GTGGTGCCAGAGGTAAGACT	GTCTCGTGGGCTCGGAGATGTGTATAAGAGACAG TCACCTAAACACGATGCCCT
OT35	TCGTCGGCAGCGTCAGATGTGTATAAGAGACAG GTAGATACTACTAGGGCTCACT	GTCTCGTGGGCTCGGAGATGTGTATAAGAGACAG CAGACTGGCAGGAGAAGAGA
OT36	TCGTCGGCAGCGTCAGATGTGTATAAGAGACAG CTGGGCACATGGGAATGATG	GTCTCGTGGGCTCGGAGATGTGTATAAGAGACAG GGAATTGCACAGTGACCCTT
OT37	TCGTCGGCAGCGTCAGATGTGTATAAGAGACAG CAGACACTCAACGCCATCAG	GTCTCGTGGGCTCGGAGATGTGTATAAGAGACAG CCCATGCAAGTCTGATGTCC
OT38	TCGTCGGCAGCGTCAGATGTGTATAAGAGACAG TGGGTGGTGCTGTGAACTAT	GTCTCGTGGGCTCGGAGATGTGTATAAGAGACAG AGGTATGCTGTCTTCTAGGTGA
OT39	TCGTCGGCAGCGTCAGATGTGTATAAGAGACAG TGAACAGTCATACCACCAAGTAA	GTCTCGTGGGCTCGGAGATGTGTATAAGAGACAG CAGCCAGTTTGATGACAGCA
OT40	TCGTCGGCAGCGTCAGATGTGTATAAGAGACAG TGCTTCTCAATATTCCCCAGC	GTCTCGTGGGCTCGGAGATGTGTATAAGAGACAG GGCCTCCAAAGTTCAGGGA
OT41	TCGTCGGCAGCGTCAGATGTGTATAAGAGACAG CCAACCACACTTCCCTCTCT	GTCTCGTGGGCTCGGAGATGTGTATAAGAGACAG CACCATAGGCACAAAGGTAGC
OT42	TCGTCGGCAGCGTCAGATGTGTATAAGAGACAG ACTGTACCATAACGATGCTAGC	GTCTCGTGGGCTCGGAGATGTGTATAAGAGACAG CCTTGATTCCGCCTTCTTTGT
OT43	TCGTCGGCAGCGTCAGATGTGTATAAGAGACAG GCCATGCCTAAAAGAAGCCA	GTCTCGTGGGCTCGGAGATGTGTATAAGAGACAG AGTTGACTTCTGTGCCCTTG
OT44	TCGTCGGCAGCGTCAGATGTGTATAAGAGACAG ATAGCAAGTGTGAAGGCCCA	GTCTCGTGGGCTCGGAGATGTGTATAAGAGACAG TGCTTCCAAACATCCCTTCC
OT45	TCGTCGGCAGCGTCAGATGTGTATAAGAGACAG GAGCCCTCCCATTGCCTAA	GTCTCGTGGGCTCGGAGATGTGTATAAGAGACAG AAGCACAGTTCTAGGCATATAGT
OT46	TCGTCGGCAGCGTCAGATGTGTATAAGAGACAG TAGAGATAGGCTTGGCAGGG	GTCTCGTGGGCTCGGAGATGTGTATAAGAGACAG AACTCGAATGCTCAACTGGC
OT47	TCGTCGGCAGCGTCAGATGTGTATAAGAGACAG AGGAAGATCACTGTCACCGT	GTCTCGTGGGCTCGGAGATGTGTATAAGAGACAG TCCCCTGTAATCCACTCACT
OT48	TCGTCGGCAGCGTCAGATGTGTATAAGAGACAG TGTCCTCCACTTCATTTCTTCAG	GTCTCGTGGGCTCGGAGATGTGTATAAGAGACAG ACCCTCTTCATTTCCCCTGG
OT49	TCGTCGGCAGCGTCAGATGTGTATAAGAGACAG GCAAGGAAGTGTTGTTCAGC	GTCTCGTGGGCTCGGAGATGTGTATAAGAGACAG TCTGACTCGTCTTTCCACAGA
OT50	TCGTCGGCAGCGTCAGATGTGTATAAGAGACAG GACTCTGGACTTGATGCCCT	GTCTCGTGGGCTCGGAGATGTGTATAAGAGACAG CCCACAGGCTCAACACTACA
OT51	TCGTCGGCAGCGTCAGATGTGTATAAGAGACAG CCACACCATGATCTGATTGCA	GTCTCGTGGGCTCGGAGATGTGTATAAGAGACAG GTCATCATCTCTGTTTCCGCT
OT52	TCGTCGGCAGCGTCAGATGTGTATAAGAGACAG AGGGTTAAGTGAGCAAGCAATG	GTCTCGTGGGCTCGGAGATGTGTATAAGAGACAG CCATGACTGCAATTCCACTGA
OT53	TCGTCGGCAGCGTCAGATGTGTATAAGAGACAG ACTCTACAGGGAAAACAGAACCA	GTCTCGTGGGCTCGGAGATGTGTATAAGAGACAG AAAGGTGCCTGTCAGTGGG
OT54	TCGTCGGCAGCGTCAGATGTGTATAAGAGACAG TGACAGAAAGAGGAGGAGACA	GTCTCGTGGGCTCGGAGATGTGTATAAGAGACAG CTCTGAAGGCTCTTGGGGAT
OT55	TCGTCGGCAGCGTCAGATGTGTATAAGAGACAG GGCACTTCTACATACAATGACT	GTCTCGTGGGCTCGGAGATGTGTATAAGAGACAG TCATTTTGGAGTCAGATTTAGGT
OT56	TCGTCGGCAGCGTCAGATGTGTATAAGAGACAG GGCATCACTCAGCTCTAAGG	GTCTCGTGGGCTCGGAGATGTGTATAAGAGACAG ACCAATCCCTCTGCACACA
OT57	TCGTCGGCAGCGTCAGATGTGTATAAGAGACAG GAGCGGGTCTTGAGCACC	GTCTCGTGGGCTCGGAGATGTGTATAAGAGACAG AGTATGAAATTCACATGGCCAC

MATERIALS AND METHODS

Vectors

The following adenine base editor-expressing plasmids were obtained from Addgene as a gift from Dr. Benjamin Kleinstiver:[1] SpG-ABE7.10 (pCMV-T7-ABEmax(7.10)-SpG-P2A-EGFP; Addgene plasmid # 140002; http://n2t.net/addgene:140002; RRID:Addgene_140002), SpG-ABE8.20 (pCMV-T7-ABE8.20m-nSpG-P2A-EGFP; Addgene plasmid # 185916; http://n2t.net/addgene:185916; RRID:Addgene_185916), SpG-ABE8e (pCMV-T7-ABE8e-nSpG-P2A-EGFP; Addgene plasmid # 185911; http://n2t.net/addgene:185911; RRID:Addgene_185911), and SpRY-ABE8e (pCMV-T7-ABE8e-nSpRY-P2A-EGFP; Addgene plasmid # 185912; http://n2t.net/addgene:185912; RRID:Addgene_185912). The following adenine base editor-expressing plasmid was obtained from Addgene as a gift from Dr. Nicole Gaudelli:[2] ABE8.8 (ABE8.8-m; Addgene plasmid # 136294; http://n2t.net/addgene:136294; RRID:Addgene 136294). The SpRY-ABE8.8 plasmid was made by combining elements of SpRY-ABE8e and ABE8.8 by standard molecular biology techniques. The following plasmid was obtained from Addgene as a gift from Dr. Joseph Jacobson:[3] iSpyMac (iSpyMac; Addgene plasmid # 155014; http://n2t.net/addgene:155014; RRID:Addgene 155014). The iSpyMac-ABE8.8 plasmid was made by combining elements of iSpyMac and ABE8.8 by standard molecular biology techniques. gRNAs were expressed from the pGuide plasmid (pGuide; Addgene plasmid # 64711; http://n2t.net/addgene:64711; RRID:Addgene_64711), with each of the following spacer sequences (plus an additional 5’ G to facilitate U6 expression) cloned into the plasmid: PAH1, 5’-AAGGGCCAAGGTATTGTGGC-3’; PAH2, 5’-AGGGCCAAGGTATTGTGGCA-3’; PAH3, 5’-GGGCCAAGGTATTGTGGCAG-3’; PAH4, 5’-GGCCAAGGTATTGTGGCAGC-3’; PAH5, 5’-GCCAAGGTATTGTGGCAGCA-3’; and PAH6, 5’-CCAAGGTATTGTGGCAGCAA-3’. pmaxGFP was purchased from Lonza. Any of the newly generated plasmids is readily available from the authors via a Transfer of Research Material agreement with the University of Pennsylvania.

RNA Production

Guide RNAs were chemically synthesized under solid phase synthesis conditions by a commercial supplier (Agilent) with end-modifications as well as heavy 2’-O-methylribosugar modification as previously described.[4] 100-mer PAH4 gRNA: 5’-mG*mG*mC*CAAGGUAUUGUGGCAGCGUUUUAGAmGmCmUmAmGmAmAmAmUmAmGmCAAGUUAAAAUAAGGCUAGUCCGUUAUCAmAmCmUmUmGmAmAmAmAmAmGmUmGmGmCmAmCmCmGmAmGmUmCmGmGmUmGmCmU*mU*mU*mU-3’, where “m” and * respectively indicate 2’-O-methylation and phosphorothioate linkage. 120-mer P56 pegRNA: 5’-mA*mC*mU*UUGCUGCCACAAUACCUGUUUUAGAmGmCmUmAmGmAmAmAmUmAmGmCAAGUUAAAAUAAGGCUAGUCCGUUAUCAmAmCmUmUmGmAmAmAmAmAmGmUmGmGmCmAmCmCmGmAmGmUmCmGmGmUmGmCGAGAAGGGACGUGGUAUUGUG*mG*mC*mA-3’. 100-mer N19 ngRNA: 5’-mU*mG*mA*GAAGGGACGUGGUAUUGGUUUUAGAmGmCmUmAmGmAmAmAmUmAmGmCAAGUUAAAAUAAGGCUAGUCCGUUAUCAmAmCmUmUmGmAmAmAmAmAmGmUmGmGmCmAmCmCmGmAmGmUmCmGmGmUmGmCmU*mU*mU*mU-3’. SpRY-ABE8.8, PEmax,[5] and GFP mRNAs were produced via in vitro transcription (IVT) and purification. In brief, a plasmid DNA template containing a codon-optimized gene coding sequence and a 3’ polyadenylate sequence was linearized. An IVT reaction containing linearized DNA template, T7 RNA polymerase, NTPs, and cap analog was performed to produce mRNA containing N1-methylpseudouridine. After digestion of the DNA template with DNase I, the mRNA product underwent purification and buffer exchange, and the purity of the final mRNA product was assessed with spectrophotometry and capillary gel electrophoresis. Elimination of double-stranded RNA contaminants was assessed using dot blots and transfection into human dendritic cells.

Endotoxin content was measured using a chromogenic Limulus amebocyte lysate (LAL) assay; all assays were negative.

LNP Formulation

LNPs were formulated as previously described,[4,6] with the lipid components (SM-102, 1,2-distearoyl-sn-glycero-3-phosphocholine, cholesterol, and PEG-2000 at molar ratios of 50:10:38.5:1.5) being rapidly mixed with an aqueous buffer solution containing (1) SpRY-ABE8.8 mRNA and PAH4 gRNA in a 1:1 ratio by weight, (2) PEmax mRNA, P56 pegRNA, and N19 ngRNA in a 3:2:1 ratio by weight, or (3) GFP mRNA in 25 mM sodium acetate (pH 4.0), with an N:P ratio of 5.6. The resulting LNP formulation was subsequently dialyzed against sucrose-containing buffer, concentrated using Amicon Ultra-15 mL Centrifugal Filter Units (Millipore Sigma), sterile-filtered using 0.2-μm filters, and frozen until use.

Culture and Transfection of HuH-7 Cells

The methods were similar to those previously described.[4] *PAH* c.1222C>T homozygous HuH-7 cell lines were generated as described elsewhere.[7] HuH-7 cells were maintained in Dulbecco's modified Eagle's medium (containing 4 mM L-glutamine and 1 g/L glucose) with 10% fetal bovine serum and 1% penicillin/streptomycin at 37°C with 5% CO_2. HuH-7 cells were seeded on 6-well plates (Corning) at 3.5×10^5 cells per well. At 16–24 hours after seeding, cells were transfected at approximately 80–90% confluency with 9 µL TransIT®-LT1 Transfection Reagent (MIR2300, Mirus), 2 µg base editor plasmid, and 1 µg gRNA plasmid per well according to the manufacturer's instructions; alternatively, GFP-expressing pmaxGFP plasmid was transfected. LNPs were added at various doses (quantified by the total amount of RNA within the LNPs) directly to the media. Cells for base editing experiments were cultured for 72 hours after transfection, and then media were removed, cells were washed with 1× DPBS (Corning), and genomic DNA was isolated using the DNeasy Blood and Tissue Kit (QIAGEN) according to the manufacturer's instructions. GFP-expressing cells were cultured for 48 hours after transfection, and then the cells were trypsinized and resuspended into 200 µL 1× DPBS with 0.1% BSA; flow cytometry for GFP positivity was performed on a CytoFLEX S Flow Cytometer (Beckman Coulter) and quantified with FlowJo v10.9.

Generation of Humanized PKU Mouse Model

The methods were similar to those previously described.[4] The PKU mouse model with humanized *Pah* c.1222C>T alleles was generated using in vitro transcribed Cas9 mRNA, a synthetic gRNA (spacer sequence 5'-AGCGAACGGAGAAGGGCCGG-3') (Synthego), and a synthetic single-strand DNA oligonucleotide (Integrated DNA Technologies) with homology arms matching the target site and harboring the c.1222C>T variant and synonymous variants (bold with underline): 5'-AAAAGCCACTTGGAACTCCTCCAGGATAACCTGTCTTTAAATGGTGTCCTTCACTGGGGTCCTTGGTTTTGGTTTCAGGA**AC**TTTGCTGCCACAAT**A**CC**TT**GGCCCTTCTC**A**GTTCGCTA**C**GACCCCTACACTCAAAGGGTTGAGGTCCTGGACAATACTCAGCAGTTGAAGATTTTAGCTGACTCCATTAATAGTAAGT-3'. The mixture of the 3 components was injected into cytoplasm of fertilized oocytes from C57BL/6J mice at the Penn Vet Transgenic Mouse Core (https://www.vet.upenn.edu/research/core-resources-facilities/transgenic-mouse-core). Genomic DNA samples from founders were screened for knock-in of the desired sequence in the *Pah* locus via homology-directed repair. Founders with the humanized c.1222C>T allele were bred through two generations to obtain homozygous mice. This humanized PKU mouse model is readily available from the authors via a Transfer of Research Material agreement with the University of Pennsylvania.

Mouse Studies

The methods were similar to those previously described.[4] All procedures used in mouse studies were approved by the Institutional Animal Care and Use Committee at the University of Pennsylvania (protocol #805887), where the studies were performed, and were consistent with local, state, and federal regulations as applicable, including the National Institutes of Health Guide for the Care and Use of Laboratory Animals. Mice were maintained on a 12-hour light/12-hour dark cycle, with a temperature range of 65°F to 75°F and a humidity range of 40% to 60%, and were fed ad libitum with a chow diet (LabDiet, Laboratory Autoclavable Rodent Diet 5010). Homozygous humanized PKU mice, as well as heterozygous humanized non-PKU mice, were generated as littermates/colonymates via timed breeding, in some cases using wild-type C57BL/6J mice (stock no. 000664; RRID:IMSR_JAX:000664) obtained from The Jackson Laboratory. Genotyping was performed using PCR amplification from genomic DNA samples (prepared from clipped tails/ears) followed by Sanger sequencing or by next-generation sequencing. Age-matched female and male colonymates were used for experiments at approximately 8 weeks of age with random assignment of animals to various experimental groups when applicable, and with collection and analysis of data performed in a blinded fashion when possible. LNPs were administered to the mice at either 5 mg/kg or 2.5 mg/kg doses, diluted with Dulbecco's phosphate-buffered saline, via retro-orbital injection under anesthesia with 1%-2% inhaled isoflurane; similar volumes of Dulbecco's phosphate-buffered saline were administered to vehicle control mice. In short-term studies, mice were euthanized at 1 week after treatment, and 8 liver samples (2 from each lobe) were obtained on necropsy and processed with the DNeasy Blood and Tissue Kit (QIAGEN) as per the manufacturer's instructions to isolate genomic DNA. Euthanasia in all instances was achieved via terminal inhalation of carbon dioxide followed by secondary euthanasia through cervical dislocation or decapitation, consistent with the 2020 American Veterinary Medical Association Guidelines on Euthanasia. Next-generation sequencing results from the liver samples were averaged to provide quantification of whole-liver editing. Blood samples were collected via the tail tip at various timepoints (pre-treatment, day 1, day 2, day 3, day 4 or day 7), with daily measurements in the early afternoon to account for diurnal variation in blood phenylalanine levels, or via retro-orbital bleeding with capillary tubes at various timepoints (pre-treatment, 4 hours, and 24 hours) to assess cytokine and chemokine levels.

Measurement of Blood Analytes

Some methods were similar to those previously described.[4] The blood phenylalanine levels were measured by an enzymatic method using the Phenylalanine Assay Kit (MAK005, Millipore Sigma) according to the manufacturer's instructions. Plasma samples were deproteinized with a 10 kDa MWCO spin filter (CLS431478-25EA, Millipore Sigma) and pre-treated with 5 µL of tyrosinase for 10 minutes at room temperature prior to start of the assay. Reaction mixes were made according to the manufacturer's instructions, and the fluorescence intensity of each sample was measured (λ_{ex} = 535/λ_{em} = 587 nm). Aspartate aminotransferase (AST) (ab263882, Abcam) and alanine aminotransferase (ALT) (MAK052-1KT, Millipore Sigma) activity were measured according to the manufacturers' instructions. AST measurements, determined in units of pg/mL, were converted to units of IU/L via normalization to reference samples. The expression profile of cytokines and chemokines in mouse serum samples was determined using the BioLegend anti-virus response panel [13-plex bead-based assay for the quantification of interferons (α, β, γ), interleukins (1β, 6, 10, 12p70), and chemokines (MCP-1, RANTES, CXCL-1, IP-10, TNF-α, and GM-CSF)] according to the manufacturer's instructions. Briefly, kit components were thawed and reconstituted as needed. Standards were prepared by 1:4 serial dilution in assay buffer. Serum samples were diluted 1:3 in Matrix A. Filter bottom plates were prewetted with wash buffer before addition of standards and samples. 25 µL of each standard and sample was added to

the plates in duplicates and mixed with 25 µL of premixed beads solution. Plates were incubated with shaking at 500 rpm for 2 hours and washed twice, followed by addition of 25 µL detection antibodies and incubation at 500 rpm for 1 hour. Without washing, 25 µL SA-PE was added to each well and incubated with shaking at 500 rpm for 30 minutes. Plates were washed twice, and beads were resuspended in 150 µL wash buffer, transferred to FACS tubes, and acquired on an BD LSR flow cytometer. The standard curve was used to determine the concentrations of each analyte in the serum samples using a 5PL fitting.

In Silico Nomination of Candidate Off-target Sites

Sites with up to 2 mismatches, or up to 1 mismatch plus up to 2 DNA or RNA bulges, compared to the *PAH* on-target site (i.e., compared to the PAH4 protospacer sequence without any regard for PAM sequence) were identified with Cas-Designer v1.2.[8] The results of next-generation sequencing (see below) for these sites are listed in Table S2.

Next-generation Sequencing

The methods were similar to those previously described.[4] PCR reactions were performed using NEBNext Polymerase (NEB) using the primer sets listed in Table S3, designed with Primer3 v4.1.0 (https://primer3.ut.ee/). The following program was used for all genomic DNA PCRs: 98°C for 20 seconds, 35× (98°C for 20 seconds, 57°C for 30 seconds, 72°C for 10 seconds), 72°C for 2 minutes. PCR products were visualized via capillary electrophoresis (QIAxcel, QIAGEN) and then purified and normalized via an NGS Normalization 96-Well Kit (Norgen Biotek Corporation). A secondary barcoding PCR was conducted to add Illumina barcodes (Nextera XT Index Kit V2 Set A and/or Nextera XT Index Kit V2 Set D) using ≈15 ng of first-round PCR product as template, followed by purification and normalization. Final pooled libraries were quantified using a Qubit 3.0 Fluorometer (Thermo Fisher Scientific) and then after denaturation, dilution to 10 pM, and supplementation with 15% PhiX, underwent single-end or paired-end sequencing on an Illumina MiSeq System. The amplicon sequencing data were analyzed with CRISPResso2 v2[9] and scripts to quantify editing. For on-target editing, A-to-G editing was quantified at the site of the c.1222C>T variant (position 5 of the PAH4 protospacer sequence) and at the site of the potential bystander adenines (positions –2, –1, 6, and 10 of the PAH4 protospacer sequence). For candidate off-target sites, A-to-G editing was quantified throughout the editing window (positions 1 to 10 of the protospacer sequence).

Data Analysis

GFP positivity and sequencing data were analyzed as described above. Other data were collected and analyzed using GraphPad Prism v10.0.2.

References

1. Walton, R.T., Christie, K.A., Whittaker, M.N., and Kleinstiver, B.P. (2020). Unconstrained genome targeting with near-PAMless engineered CRISPR-Cas9 variants. Science *368*, 290–296. https://doi.org/10.1126/science.aba8853.

2. Gaudelli, N.M., Lam, D.K., Rees, H.A., Solá-Esteves, N.M., Barrera, L.A., Born, D.A., Edwards, A., Gehrke, J.M., Lee, S.J., Liquori, A.J., et al. (2020). Directed evolution of adenine base editors with increased activity and therapeutic application. Nat. Biotechnol. *38*, 892–900. https://doi.org/10.1038/s41587-020-0491-6.

3. Chatterjee, P., Lee, J., Nip, L., Koseki, S.R.T., Tysinger, E., Sontheimer, E.J., Jacobson, J.M., and Jakimo, N. (2020). A Cas9 with PAM recognition for adenine dinucleotides. Nat. Commun. *11*, 2474. https://doi.org/10.1038/s41467-020-16117-8.

4. Brooks, D.L., Carrasco, M.J., Qu, P., Peranteau, W.H., Ahrens-Nicklas, R.C., Musunuru, K., Alameh, M.-G., and Wang, X. (2023). Rapid and definitive treatment of phenylketonuria in variant-humanized mice with corrective editing. Nat. Commun. *14*, 3451. https://doi.org/10.1038/s41467-023-39246-2.

5. Chen, P.J., Hussmann, J.A., Yan, J., Knipping, F., Ravisankar, P., Chen, P.F., Chen, C., Nelson, J. W., Newby, G.A., Sahin, M., et al. (2021). Enhanced prime editing systems by manipulating cellular determinants of editing outcomes. Cell *184*, 5635–5652.e29. https://doi.org/10.1016/j.cell.2021.09.018.

6. Carrasco, M.J., Alishetty, S., Alameh, M.G., Said, H., Wright, L., Paige, M., Soliman, O., Weissman, D., Cleveland, T.E., 4th, Grishaev, A., et al. (2021). Ionization and structural properties of mRNA lipid nanoparticles influence expression in intramuscular and intravascular administration. Commun. Biol. *4*, 956. https://doi.org/10.1038/s42003-021-02441-2.

7. Brooks, D.L., Whittaker, M.N., Qu, P., Musunuru, K., Ahrens-Nicklas, R.C., and Wang, X. (2023). Efficient in vivo prime editing corrects the most frequent phenylketonuria variant, associated with high unmet medical need. Am. J. Hum. Genet. https://doi.org/10.1016/j.ajhg.2023.10.005.

8. Bae, S., Park, J., and Kim, J.S. (2014). Cas-OFFinder: a fast and versatile algorithm that searches for potential off-target sites of Cas9 RNA-guided endonucleases. Bioinformatics *30*, 1473–1475. https://doi.org/10.1093/bioinformatics/btu048.

9. Clement, K., Rees, H., Canver, M.C., Gehrke, J.M., Farouni, R., Hsu, J.Y., Cole, M.A., Liu, D.R., Joung, J.K., Bauer, D.E., et al. (2019). CRISPResso2 provides accurate and rapid genome editing sequence analysis. Nat. Biotechnol. *37*, 224–226. https://doi.org/10.1038/s41587-019-0032-3.
